# Synthesis, characterization, and photophysical properties of novel 9‑phenyl-9-phosphafluorene oxide derivatives

**DOI:** 10.3762/bjoc.20.274

**Published:** 2024-12-30

**Authors:** Shuxian Qiu, Duan Dong, Jiahui Li, Huiting Wen, Jinpeng Li, Yu Yang, Shengxian Zhai, Xingyuan Gao

**Affiliations:** 1 College of Chemistry and Material Science, Guangdong University of Education, Guangzhou 510303, China; 2 College of Chemistry and Chemical Engineering, Tarim University, Aral City 843300, China; 3 College of Chemistry & Environmental Engineering, Anyang Institute of Technology, Anyang 455000, China

**Keywords:** carbazole, D−A−D type, noble-metal-free system, 9‑phenyl-9-phosphafluorene oxide, photophysical properties

## Abstract

A novel series of D−A−D-type 9-phenyl-9-phosphafluorene oxide (PhFlOP) derivatives was prepared and is reported herein. The synthetic protocol involved 5 steps from commercially available 2-bromo-4-fluoro-1-nitrobenzene, featuring a noble-metal-free system, mild reaction conditions, and a good yield, especially for the final Cs_2_CO_3_-facilitated nucleophilic substitution (77–91% yield). The characterization data obtained from IR and NMR spectroscopy (^1^H, ^13^C, ^19^F, and ^31^P) as well as HRMS spectrometry were in full agreement with the expected structures, and single-crystal X-ray diffraction analysis was conducted to confirm the structure of compound **7-H**. Moreover, the photophysical properties of these PhFlOP derivatives were determined by UV–vis absorption and photoluminescence studies, revealing that their photophysical behavior can be affected by the different substituents in the donor carbazole group.

## Introduction

π-Conjugated molecular materials containing phosphine oxide (PO) groups have recently received considerable attention for their high thermal stability and unique optoelectronic features, and thus being widely applied in organic light-emitting diodes (OLEDs) [1–2]. To date, tremendous efforts have been devoted to the development of a variety of high-performing PO-based luminescent molecules [3–21] due to the benign electron injection/transport capability of PO-containing groups. Among them, 9-phenyl-9-phosphafluorene oxide (PhFlOP) is one of the most popular core units [22–26]. Compared to the traditional PO-containing moieties, PhFlOP possesses an enhanced rigid structure to reduce the possibility of nonradiative decay processes, which would improve optoelectronic properties [17,27].

Thermally activated delayed fluorescence (TADF) materials and devices have emerged rapidly in recent years, and they are mostly based on purely organic electron donor−electron acceptor (D−A) or D−A−D systems with significant intramolecular charge transfer interactions for frontier molecular orbital separation [28–30]. Due to the electron-accepting properties, PhFlOP can clearly act as an acceptor group in TADF emitters, indicating great potential for the development of highly efficient TADF molecules. In 2019, Nishida and co-workers prepared 5 D–A–D-type PhFlOP derivatives with electron-donating diarylamine or carbazole moieties in positions 2 and 8. They conducted optical and electrochemical studies, showing that the photophysical properties of PhFlOP depend on the nature of the electron-donating groups [31]. Later, Wu and co-workers introduced various electron donors to the PhFlOP unit to form new TADF emitters with high electroluminescence efficiency [32–33].

Despite this progress, TADF emitters containing the PhFlOP unit as an electron acceptor are still scarce. Meanwhile, the syntheses of the TADF emitters by the groups of Nishida and Wu both utilized palladium noble metal as a catalyst [31–33]. Therefore, it is of great significance to develop cost-effective synthetic access to PhFlOP-based TADF emitters. Additionally, the design of TADF emitters with the PhFlOP acceptor moiety and the carbazole donor moiety is lacking structural diversity. Herein, we present a 5-step synthesis of several novel D−A−D-type PhFlOP derivatives with substituted carbazole groups as donors, starting from commercially available 2-bromo-4-fluoro-1-nitrobenzene under noble-metal-free conditions. The structures and photophysical properties of the desired molecules were also determined.

## Results and Discussion

### Synthesis and structural characterization

**Scheme 1:**
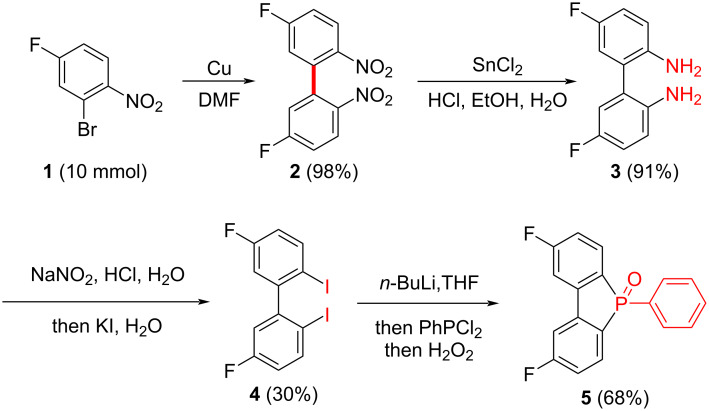
Preparation of key intermediate **5**.

**Scheme 2:**
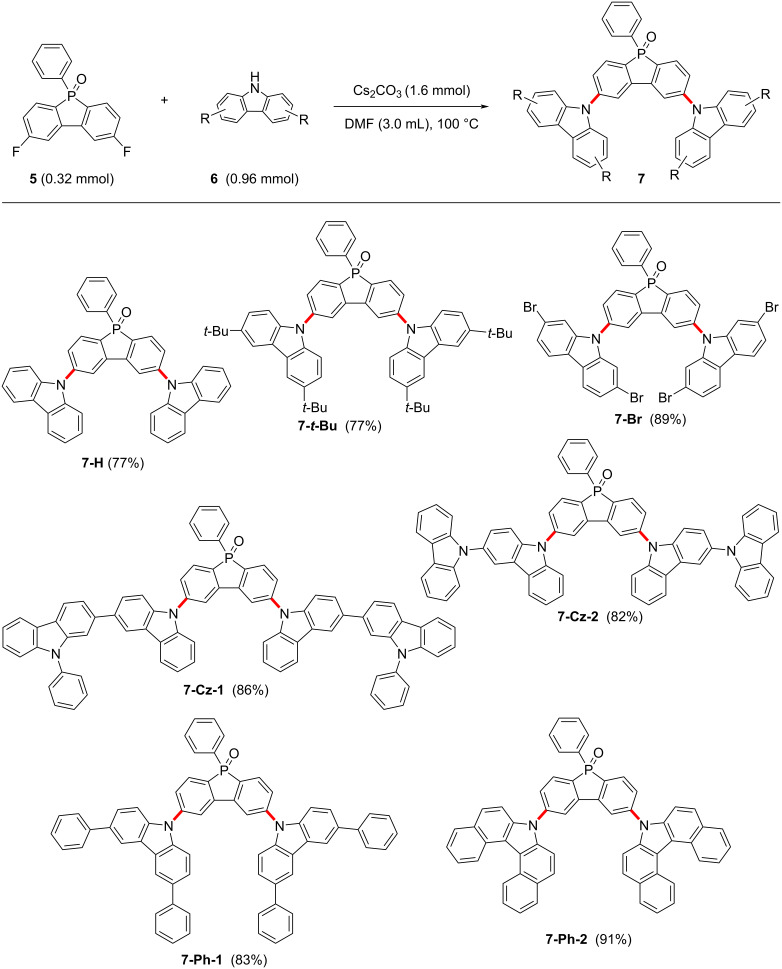
Synthesis of PhFlOP-based molecules **7**.

The synthesis of the PhFlOP-based compounds **7** was achieved in 5 steps starting from commercially available 2-bromo-4-fluoro-1-nitrobenzene (**1**, Scheme 1 and Scheme 2). For the preparation of the key intermediate **5** (Scheme 1), self-coupling of **1** in the presence of copper followed by reduction of the nitro group generated diamine compound **3** (89% yield over 2 steps) [34]. Upon exposure to NaNO_2_/HCl, diamine **3** was transformed into a diazonium salt, which was captured by KI to deliver the diiodide **4**. Treatment of **4** with *n*-BuLi, PhPCl_2_, and H_2_O_2_ sequentially gave 2,8-difluoro-5-phenylbenzo[*b*]phosphindole 5-oxide (**5**) in 68% yield.

With compound **5** in hand, we turned our attention to the synthesis of PhFlOP-based compounds through a Cs_2_CO_3_-facilitated nucleophilic substitution with substituted carbazoles as the nucleophiles (Scheme 2). For example, *tert*-butyl, bromo, carbazolyl, or phenyl substituents were introduced into the carbazoles. To our delight, by treatment of **5** with substituted carbazoles **6** in the presence of Cs_2_CO_3_ (5.0 equiv) in DMF at 100 °C, seven 2,8-bis(9*H*-carbazol-9-yl)-5-phenylbenzo[*b*]phosphindole 5-oxide derivatives **7** were furnished in good to excellent yields (77–91%). The structural characterization of the obtained molecules **2**–**7** was performed by NMR spectroscopy, which confirmed the synthetic outcomes (Figures S1–S11, Supporting Information File 1). The structures of compounds **7** were further confirmed by HRMS and IR analyses (Figures S12–S18, Supporting Information File 1).

**Figure 1:**
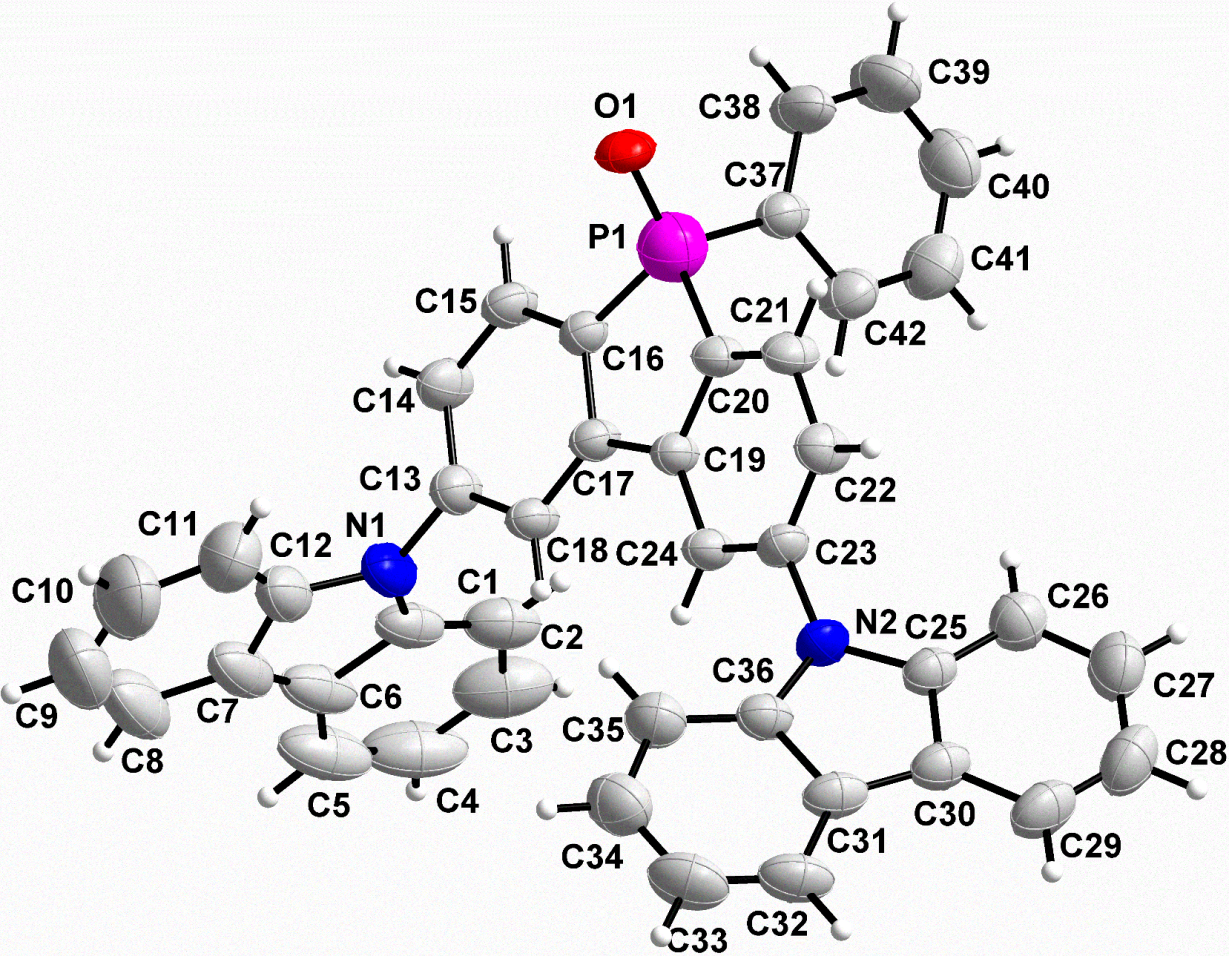
An ORTEP drawing obtained using the X-ray crystallographic data of **7-H**.

In addition, the chemical structure of **7-H** was fully elucidated by single-crystal X-ray crystallography, which was performed on a Bruker APEX-II CCD diffractometer using graphite monochromated Mo *K*α radiation at a temperature of 296 ± 2 K. Crystallographic data were deposited with the Cambridge Crystallographic Data Centre under accession number CCDC 2256875. The crystallographic details are summarized in Table 1, and the structure of **7-H** is shown in Figure 1 as an ORTEP diagram.

**Table 1:** Crystal data and structural parameters for **7-H**.

parameter	**7-H**

empirical formula	C_42_H_27_N_2_OP
Fw	606.19
temperature (K)	296(2)
crystal system	monoclinic
space group	*P*2(1)/*c*
*a* (Å)	13.886(3)
*b* (Å)	17.477(4)
*c* (Å)	15.239(3)
α (deg)	90
β (deg)	105.503(4)
γ (deg)	90
volume (Å^3^)	3563.7(13)
*Z*	4
ρ calcd (mg/m^3^)	1.586
μ (Mo *K*α, mm^−1^)	0.612
*F*(000)	1702
number of reflections	26109
unique reflections	6289
data/restraints/parameters	8850/0/437
*R*_int_	0.0253
GOF (*F*^2^)	1.062
completeness to θ = 25.242	99.8%
final *R* indices [*I* > 2σ(*I*)]	*R*1 = 0.0769, *wR*2 = 0.2894

### Photophysical properties

**Figure 2:**
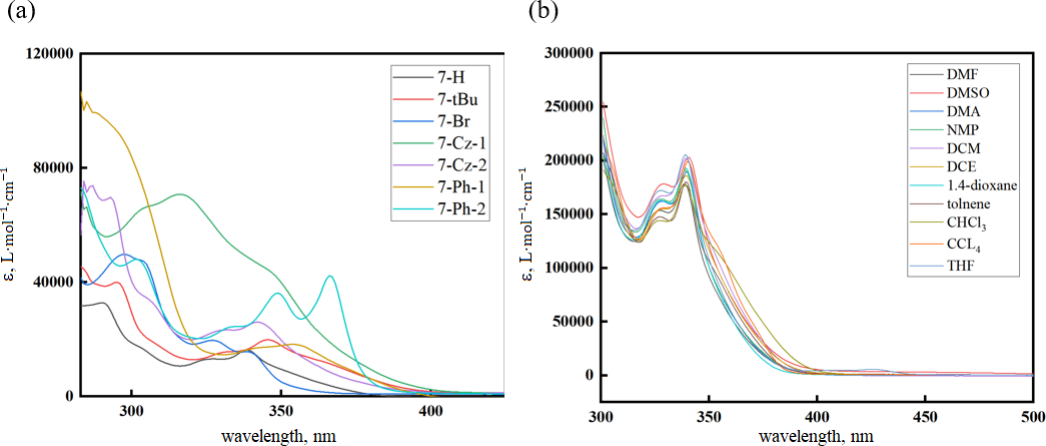
(a) UV–vis absorption spectra of the PhFlOP-based emitters **7** measured at a concentration of ≈10^−5^ M in toluene at room temperature. (b) UV–vis absorption spectra of **7-H** measured at a concentration of ≈10^−4^ M in different solvents at room temperature.

In order to investigate the photophysical properties of the PhFlOP-based molecules **7**, UV−vis absorption and photoluminescence (PL) studies were conducted. UV−vis absorption spectra of **7** in toluene solution at room temperature are shown in Figure 2, and the corresponding data are included in Table 2. The spectra in Figure 2a exhibit two major absorption bands at ≈290 nm and ≈340 nm. The band at around 290 nm might be induced by π→π* transitions associated with the conjugated system, while the band at around 340 nm is attributed to intramolecular charge transfer processes. The low-energy absorption bands of **7-*t*-Bu** (λ_max_ = 345 nm, Table 2) and **7-Cz-2** (λ_max_ = 342 nm) are slightly redshifted compared to **7-H** (λ_max_ = 338 nm), and larger redshifts are observed for **7-Ph-1** (λ_max_ = 354 nm) and **7-Ph-2** (λ_max_ = 366 nm). In contrast to **7-H**, **7-Br** (λ_max_ = 327 nm) and **7-Cz-1** (λ_max_ = 316 nm) show a blueshift. With a stronger electron-donating ability than **7-Cz-1**, **7-Cz-2** shows a lower energy level for the absorption band stemming from intramolecular charge transfer, as indicated by the λ_max_ value of 342 nm. In addition, the effect of solvent polarity on the UV−vis absorption was studied with **7-H** (Figure 2b). The spectra show that there is no significant difference in the absorption bands in different solvents, indicating that the polar environment has insignificant effect on the molecular ground state of **7-H**.

**Table 2:** Photophysical data of the PhFlOP-based emitters **7**.

compound	λ_abs_, nm (log ε)^a^	λ_em_, nm^b^	PLQY^c^	τ_DF_ (ms)^d^

**7-H**	290 (4.52), 338 (4.21) [291 (4.55), 338 (4.31)]^e^	408 (412, 450, 478)^f^	0.32 (0.16)	1.94 (296)
**7-** ***t*** **-Bu**	295 (4.60), 345 (4.30)	424	0.25	1.23
**7-Br**	298 (4.70), 327 (4.29)	383	0.22	0.88
**7-Cz-1**	285 (4.82), 316 (4.85)	436	0.38	1.23
**7-Cz-2**	293 (4.84), 342 (4.41)	444	0.31	1.15
**7-Ph-1**	285 (5.01), 354 (4.26)	425	0.34	1.49
**7-Ph-2**	302 (4.68), 349 (4.56), 366 (4.63)	392	0.27	1.17

^a^Measured at a concentration of ≈10^−5^ M in toluene at room temperature. ^b^Measured in toluene at room temperature. ^c^The absolute PL quantum yield (PLQY) was measured in degassed toluene at room temperature using an integrating sphere, and the reported PLQY of solid **7-H** is presented in parentheses [31]. ^d^The delayed fluorescence lifetime (τDF) was measured in degassed toluene at room temperature, and the reported τDF of **7-H** in toluene at 77 K is presented in parentheses [31]. ^e^Reported data are presented in square brackets [31]. ^f^The values in parentheses are reported λ_em_ in various solvents, namely toluene, DCM, and CH_3_CN [31].

**Figure 3:**
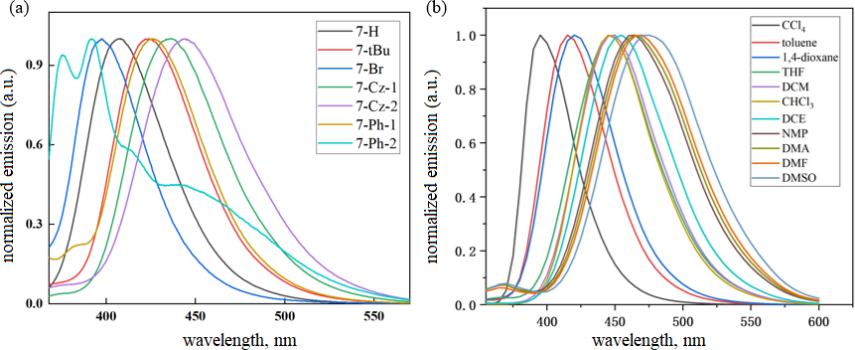
(a) PL spectra of the PhFlOP-based emitters **7** measured in toluene at room temperature. (b) PL spectra of **7-H** measured in different solvents at room temperature.

The PL spectra of the PhFlOP-based compounds **7** in toluene at room temperature are shown in Figure 3, and the λ_em_ values are included in Table 2. Different emission wavelengths are observed due to the various substituents present in the donor carbazole group (Figure 3a). Compared to **7-H** (λ_em_ = 408 nm, Table 2), compounds **7-*t-*Bu** (λ_em_ = 424 nm), **7-Cz-1** (λ_em_ = 436 nm), **7-Cz-2** (λ_em_ = 444 nm), and **7-Ph-1** (λ_em_ = 425 nm) all show a redshift due to the electron-donating groups (*t-*Bu, Cz, Ph) on the carbazole moiety. However, **7-Ph-2** exhibits a significantly blueshifted emission maximum at 392 nm, perhaps as a consequence of a more rigid configuration. As for **7-Br**, owing to the electron-withdrawing properties of Br, it displays a blueshifted PL maximum at 383 nm. The emission wavelength of **7-Cz-2** has a slight redshift compared to **7-Cz-1**, which may be induced by the stronger electron-donating feature of the carbazole substituent located on the donor carbazole group. In addition, we tested the emission wavelength of **7-H** in different solvents (Figure 3b) and found that the maximum is redshifted gradually with increasing solvent polarity, which indicates the CT feature in the excited state. Further, the solvent dependence of **7-H** exhibits good consistence with that reported by the Nishida group [31]. The PLQY and τ_DF_ values of the PhFlOP-based emitters **7** were measured in degassed toluene, and the corresponding data are included in Table 2, showing a PLQY ranging from 0.22–0.38 and a τ_DF_ in the order of milliseconds.

## Conclusion

In summary, we have developed a 5-step synthesis of a series of D−A−D-type PhFlOP derivatives **7** with 2-bromo-4-fluoro-1-nitrobenzene as the starting material. This novel protocol is mild, noble-metal-free, and operationally simple. The structure of **7-H** was confirmed by single-crystal X-ray diffraction. Furthermore, UV–vis absorption and PL studies were carried out to explore the photophysical properties of these PhFlOP derivatives. Investigations for further applications of the PhFlOP-based emitters **7** are still ongoing.

## Supporting Information

File 1 General information, experimental procedures, characterization data, and copies of spectra.

## Data Availability

All data that supports the findings of this study is available in the published article and/or the supporting information of this article.
