# Supplementary material for: Synthesis, characterization, and photophysical properties of novel 9‑phenyl-9-phosphafluorene oxide derivatives
Source: Beilstein J Org Chem. 2024 Dec 30;20:3299–305. doi: 10.3762/bjoc.20.274 (PMC11702303; doi:10.3762/bjoc.20.274)

BEILSTEIN JOURNAL OF ORGANIC CHEMISTRY

Supporting Information

for

Synthesis, characterization, and photophysical properties of novel 9-phenyl-9-phosphafluorene oxide derivatives

Shuxian Qiu, Duan Dong, Jiahui Li, Huiting Wen, Jinpeng Li, Yu Yang, Shengxian Zhai and Xingyuan Gao

Beilstein J. Org. Chem. **2024,** *20,* 3299–3305. doi:10.3762/bjoc.20.274

General information, experimental procedures, characterization data, and copies of spectra

License and Terms: This is a supporting information file under the terms of the Creative Commons Attribution License (https://creativecommons.org/licenses/by/4.0). Please note that the reuse, redistribution and reproduction in particular requires that the author(s) and source are credited and that individual graphics may be subject to special legal provisions.
The license is subject to the *Beilstein Journal of Organic Chemistry* terms and conditions: (https://www.beilstein-journals.org/bjoc/terms)

Table of contents

- Materials and methods
- Synthesis of 2,8-difluoro-5-phenylbenzo[*b*]phosphindole 5-oxide (**5**)
- General procedure for the synthesis of PhFlOP-based molecules **7**
- ^{1}H, ^{13}C, ^{19}F and ^{31}P NMR spectra
- IR spectra

- **Materials and methods**

All reactions were carried out under nitrogen atmosphere with dry solvents under anhydrous conditions, unless otherwise noted. All the chemicals were purchased commercially, and used without further purification. Thin-layer chromatography (TLC) was conducted with 0.25 mm Tsingdao silica gel plates (60F-254) and visualized by exposure to UV light (254 nm) or stained with potassium permanganate. Flash column chromatography was performed on Tsingdao silica gel (200–300 mesh). ^{1}H NMR spectra were recorded on Bruker spectrometers (at 400 MHz) and reported relative to deuterated solvent signals or tetramethylsilane internal standard signals. Data for ^{1}H NMR spectra were reported as follows: chemical shift (δ/ppm), multiplicity (s = singlet, d = doublet, t = triplet, q = quartet, m = multiplet, br = broad.), coupling constant (J/Hz) and integration. ^{13}C NMR spectra were recorded on Bruker Spectrometers (100 MHz). Data for ^{13}C NMR, ^{19}F NMR, and ^{31}P NMR spectra were reported in terms of chemical shift. X-ray diffraction was performed on Bruker APEX-II CCD diffractometer using graphite monochromated Mo Kα radiation at a temperature of 296 ± 2 K. Ultraviolet−visible (UV−vis) spectra were recorded on a SHIMADU UV2600 spectrophotometer. The photoluminescent spectra were measured on a HITACHI F-7000 spectrophotometer. Lifetimes of compounds **7** were measured using an Edinburgh FLS1000 Photoluminescence Spectrometer. Absolute photoluminescence quantum yields (PLQYs) of solution samples were obtained using an integrating sphere. High-resolution mass spectrometry (HRMS) was conducted on Bruker Apex IV RTMS.

- **Synthesis of 2,8-difluoro-5-phenylbenzo[*b*]phosphindole 5-oxide (5)**

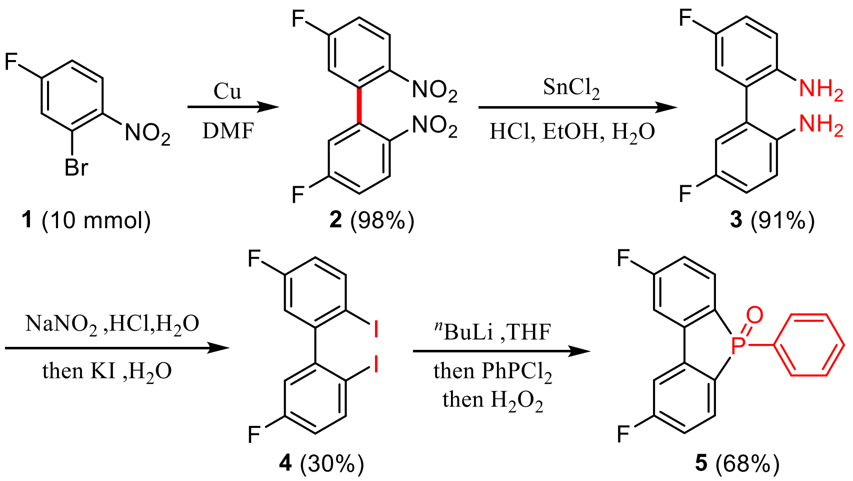

The synthesis of 5,5'-difluoro-2,2'-dinitro-1,1'-biphenyl (**2**): a solution of 2-bromo-4-fluoro-1-nitrobenzene (**1**, 2.189 g, 10 mmol, 1.0 equiv) and copper power (1.4 g, 22 mmol, 2.2 equiv) in DMF (15 mL) was stirred under nitrogen atmosphere at 125 °C for 1 h. After filtration of the hot brown residue, water (100 mL) was added and the mixture was extracted with ethyl acetate three times (20 mL × 3). The combined organic layer was washed with brine, dried over anhydrous Na_2SO_4, filtered and concentrated under reduced pressure. The residue was recrystallization from EtOAc/petroleum ether. The product **2** was obtained as yellow solid in 98% yield. ^{1}H NMR (400 MHz, $CDCl_3$) δ 8.343 (t, J = 4.4 Hz, 2H), 7.310 (s, 2H) ,7.024 (t, J = 3.6 Hz, 2H). ^{13}C NMR (100 MHz, $CDCl_3$) δ 164.8 (d, J = 258.0 Hz), 142.9, 136.4, 128.0 (d, J = 9.9 Hz), 117.8 (d, J = 24.4 Hz), 116.4 (d, J = 22.7 Hz). ^{19}F NMR (376 MHz, $CDCl_3$) δ -101.998.

The synthesis of 5,5'-difluoro-[1,1'-biphenyl]-2,2'-diamine (**3**): under nitrogen atmosphere, a solution of 5,5'-difluoro-2,2'-dinitro-1,1'-biphenyl (**2**, 2.52 g, 9 mmol, 1.0 equiv) in 22 mL of ethanol, 4.5 mL of H_2O and 9 mL of 38% aqueous HCl, then, Stannous chloride dihydrate (12.182 g, 54 mmol, 6.0 equiv) was added in batches. The mixture was stirred and refluxed for 4 h. After cooling to room temperature, NaOH solution (0.002 M) was added until the solution became alkaline. The resulting solution was extracted with ethyl acetate three times. The combined organic layer was washed with brine, dried over anhydrous Na_2SO_4, filtered and concentrated under reduced pressure. The residue was purified by column chromatography over silica (eluting with PE : EA = 10 : 1 to 3:1) to give the product **3** as light brown solid in 91%. ^{1}H NMR (400 MHz, $CDCl_3$) δ 6.917 (t, J = 7.2 Hz, 2H), 6.847 (t, J = 4.4 HZ, 2H), 6.725 (t, J = 4.0 Hz, 2H), 3.627 (br, 4H). ^{13}C NMR (100 MHz, $CDCl_3$) δ 156.2 (d, J = 235.9 Hz), 140.1, 124.8 (d, J = 7.3 Hz), 117.1 (d, J = 22.2 Hz), 116.8 (d, J = 7.8 Hz), 115.8 (d, J = 22.1 Hz). ^{19}F NMR (376 MHz, $CDCl_3$) δ -126.173.

The synthesis of 5,5'-difluoro-2,2'-diiodo-1,1'-biphenyl (**4**): 5,5'-difluoro-[1,1'-biphenyl]-2,2'-diamine (**3**, 1.1 g, 5 mmol, 1.0 equiv) was dissolved in H_2O (15 mL) and hydrochloric acid (29 mL ,12 M). At −10 °C, a solution of $NaNO_2$ (1.035 g, 15 mmol, 3.0 equiv) in H_2O (23 mL) was added dropwise and stirred for 30 min, KI (3.154 g, 19

mmol, 3.8 equiv) in water (33 mL) was added dropwise at −15 °C. After stirring for 1 h at this temperature. the resulting mixture was warmed to room temperature and stirred overnight. The reaction was quenched with saturated sodium sulfite solution, and extracted with ethyl acetate three times. The combined organic layer was washed with saturated sodium sulfite solution, brine, dried over anhydrous Na_2SO_4, filtered and concentrated under reduced pressure. The residue was purified by column chromatography over silica with petroleum ether as the eluent. The product **4** was obtained as white solid in 30% yield. ^{1}H NMR (400 MHz, $CDCl_3$) δ 7.877-7.882 (m, 2H), 6.889-6.944 (m, 4H). ^{13}C NMR (100 MHz, $CDCl_3$) δ 162.7 (d, J = 247.8 Hz), 149.4 (d, J = 7.1 Hz), 140.4 (d, J = 7.9 Hz), 117.4 (d, J = 4.6 Hz), 117.2 (d, J = 5.7 Hz), 92.2 (d, J = 3.3 Hz). ^{19}F NMR (376 MHz, $CDCl_3$) δ -113.583.

The synthesis of 2,8-difluoro-5-phenylbenzo[*b*]phosphindole 5-oxide (**5**): under nitrogen atmosphere, a solution of 5,5'-difluoro-2,2'-diiodo-1,1'-biphenyl (**4**, 0.442 g, 1 mmol, 1.0 equiv) in dry THF (10 mL) was cooled to −78 °C. Then, *n*-BuLi (2.4 mL, 2.4 mmol, 2.4 equiv) was added dropwise and stirred for 1 h at this temperature. After that, dichlorophenylphosphine (0.15 mL, 1.1 mmol, 1.1 equiv) was added dropwise. The reactive mixture was warmed to room temperature and stirred for 1.5 h. Hydrogen peroxide (0.52 mL, 5 mmol, 5.0 equiv) was added slowly under stirring. After being stirred vigorously for 30 min. The reaction was quenched with water, and extracted with ethyl acetate three times. The combined organic layer was washed with brine, dried over anhydrous Na_2SO_4, filtered and concentrated under reduced pressure. The residue was purified by column chromatography over silica (eluting with PE : EA = 2 : 1 to 1:1) to give the product **5** as white solid in 68% yield. ^{1}H NMR (400 MHz, $CDCl_3$) δ 7.699-7.710 (m, 2H),7.613-7.672 (m, 2H), 7.532 (m, 1H), 7.446 (t, J = 9.4 Hz, 4H), 7.124 (m, 2H). ^{13}C NMR (100 MHz, $CDCl_3$) δ 166.5 (d, J = 252.7 Hz) 143.7 (d, J = 22.4 Hz), 132.5 (d, J = 2.6 Hz), 132.1 (d, J = 10.1 Hz), 131.5 (d, J = 93.3 Hz), 130.7 (d, J = 51.4 Hz), 129.6 (d, J = 52.1 Hz), 128.9 (d, J = 12.7 Hz), 117.3 (dd, J = 22.4, 12.2 Hz), 109.2 (dd, J = 23.4, 11.1 Hz). ^{31}P NMR (162 MHz, $CDCl_3$) δ 30.823. ^{19}F NMR (376 MHz, $CDCl_3$) δ -104.281.

- **General procedure for the synthesis of PhFlOP-based molecules 7**

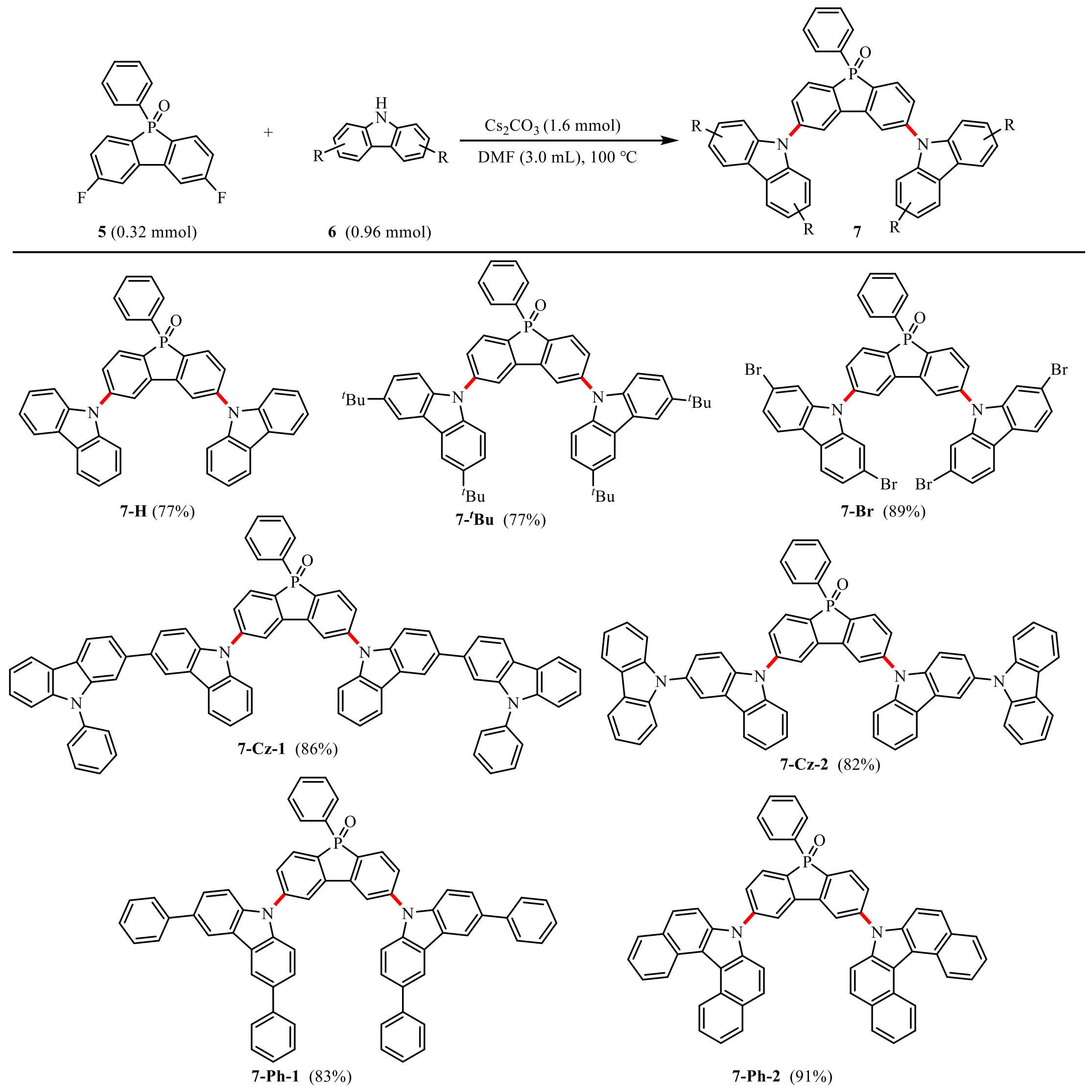

Under nitrogen atmosphere, a solution of carbazole or substituted carbazole **6** (0.96 mmol, 3.0 equiv) and Cs_2CO_3 (1.6 mmol, 5.0 equiv) in dry DMF (1.5 mL) was stirred at room temperature for 1 h. Then a solution of 2,8-difluoro-5-phenylbenzo[*b*]phosphindole 5-oxide (**5**, 0.32 mmol, 1.0 equiv) in DMF (1.5 mL) was added. The resulting mixture was stirred at 100 °C for 4 h. TLC or ^{19}F NMR was used to monitor the reaction. After cooling to room temperature, the reaction was quenched with saturated sodium chloride solution, and extracted with ethyl acetate three times. The combined organic layer was washed with brine, dried over anhydrous Na_2SO_4, filtered and concentrated under reduced pressure. The residue was purified by column chromatography over silica using a proper eluent.

7-H: 77% yield. ^{1}H NMR (400 MHz, $CDCl_3$) δ 8.134 (d, J = 5.6 Hz, 4H), 8.037 (s, 4H), 7.891 (m, 2H), 7.712 (s, 2H), 7.638 (s, 1H), 7.562 (s, 2H), 7.490 (s, 4H), 7.420 (s, 4H). ^{13}C NMR (100 MHz, $CDCl_3$) δ 143.3 (d, J = 21.8 Hz), 143.1, 140.1, 132.8, 132.3, 131.8 (d, J = 10.2 Hz), 131.4, 131.3, 129.2 (d, J = 12.5 Hz), 128.1 (d, J = 11.3 Hz), 126.4,

123. 9, 120.7 (d, J = 20.5 Hz), 119.6 (d, J = 10.0 Hz), 109.7. ^{31}P NMR (162 MHz, $CDCl_3$) δ 32.2. HRMS calculated for $C_{42}H_{28}N_2OP$ (M + H^+): 607.1934, found 607.1935.

7-*t*-Bu: 77% yield. ^{1}H NMR ($CDCl_3$, 400 MHz): δ 8.129-8.121 (d, J = 3 Hz, 4H), 8.016-7.989 (d, J = 11 Hz, 4H), 7.904-7.859 (m, 2H), 7.699 (s, 2H), 7.633-7.624 (d, J = 4 Hz, 1H), 7.552 (s, 2H), 7.458 (s, 8H), 1.450-1.444 (d, J = 2 Hz, 36H). ^{13}C NMR (100 MHz, $CDCl_3$) δ 143.8, 143.6, 138.4, 132.7, 131.7, 131.6, 131.3, 129.1 (d, J = 12.5 Hz), 127.4 (d, J = 11.5 Hz), 124.1, 123.9, 119.0 (d, J = 9.9 Hz), 116.5, 109.2, 34.8, 32.0. ^{31}P NMR (162 MHz, $CDCl_3$) δ 32.36. HRMS calculated for $C_{58}H_{60}N_2OP$ (M + H^+): 831.4438, found 831.4437.

7-Br: 89% yield. ^{1}H NMR ($CDCl_3$, 400 MHz): δ 8.073-8.048 (d, J = 10 Hz, 2H), 7.962-7.885 (m, 7H), 7.671-7.561 (m, 8H), 7.447-7.414 (m, 3H), 7.274-7.258 (m, 3H). ^{13}C NMR (100 MHz, $CDCl_3$) δ 143.23, 141.84, 141.22, 133.66, 133.09, 132.61, 132.17, 131.41, 129.36, 128.68, 124.46, 122.15, 121.76, 120.38, 120.06, 112.95. ^{31}P NMR (162 MHz, $CDCl_3$) δ 31.80. HRMS calculated for $C_{42}H_{24}Br_4N_2OP$ (M + H^+): 918.8354, found 918.8356.

7-Cz-1: 86% yield. ^{1}H NMR ($CDCl_3$, 400 MHz): δ 8.430 (s, 4H), 8.236-8.190 (m, 4H), 8.125 (s,2H), 8.042 (s, 2H), 7.923 (s, 2H), 7.788-7.718 (m, 6H), 7.620-7.538 (m, 16H), 7.497-7.419 (m, 10H), 7.344-7.281 (m, 3H). ^{13}C NMR (100 MHz, $CDCl_3$) δ 143.52, 143.30, 143.23, 141.33, 140.60, 140.09, 139.24, 137.70, 135.29, 133.96, 132.89, 132.25, 131.93, 131.43, 129.98, 129.27, 128.13, 127.52, 127.08, 126.57, 126.29, 126.16, 125.80, 124.58, 124.17, 123.99, 123.52, 120.96, 120.78, 120.48, 120.08, 119.64, 119.18, 118.97, 110.11, 110.03, 109.96, 109.86. ^{31}P NMR (162 MHz, $CDCl_3$) δ 32.33. HRMS calculated for $C_{78}H_{50}N_4OP$ (M + H^+): 1089.3717, found 1089.3715.

7-Cz-2: 82% yield. ^{1}H NMR ($CDCl_3$, 400 MHz): δ 8.294-8.285 (d, J = 4 Hz, 2H), 8.188-8.168 (d, J = 8 Hz, 6H), 8.128-8.103 (4H), 7.929 (s, 2H), 7.818-7.798 (d, J = 8 Hz, 2H), 7.712-7.679 (m, 3H), 7.585-7.561 (d, J = 10 Hz, 6H), 7.521-7.495 (m, 2H), 7.384-7.345 (m,10H), 7.330-7.283 (m, 4H). ^{13}C NMR (100 MHz, $CDCl_3$) δ 143.53, 142.85, 141.71, 140.90, 139.27, 133.00, 132.13, 131.39, 130.73, 129.30, 128.51, 127.15, 125.94, 124.99, 123.44, 123.16, 121.28, 120.94, 120.37, 119.91, 119.76, 110.79, 110.01, 109.72.^{31}P NMR (162 MHz, $CDCl_3$) δ 32.25. HRMS calculated for $C_{66}H_{41}N_4OPNa$ (M + Na^+): 959.2910, found 959.2909.

7-Ph-1: 83% yield. ^{1}H NMR ($CDCl_3$, 400 MHz): δ 8.415-8.375 (m, 4H), 8.121(s, 1H), 8.081-8.014 (m, 2H), 7.945-7.890 (m, 2H), 7.7477.638 (m, 15H), 7.593-7.566 (m, 5H), 7.514-7.440 (m, 10H), 7.388-7.318 (m, 4H). ^{31}P NMR (162 MHz, $CDCl_3$) δ 32.64. HRMS calculated for $C_{66}H_{44}N_2OP$ (M + H^+): 911.3186, found 911.3175.

7-Ph-2: 91% yield. ^{1}H NMR ($CDCl_3$, 400 MHz): δ 9.202-9.173 (m, 4H), 8.115 (s, 2H), 8.063(s, 3H), 8.017-7.953 (m, 7H), 7.849-7.831 (d, J = 7 Hz, 3H), 7.760-7.684 (m, 8H), 7.634-7.608 (m, 4H), 7.546-7.499 (m, 4H). HRMS calculated for $C_{58}H_{35}N_2OPNa$ (M + Na^+): 829.2379, found 829.2384.

- ^{1}H, ^{13}C, ^{19}F and ^{31}P NMR spectra

Figure S1 ^{1}H, ^{13}C and ^{19}F-NMR Compound 2

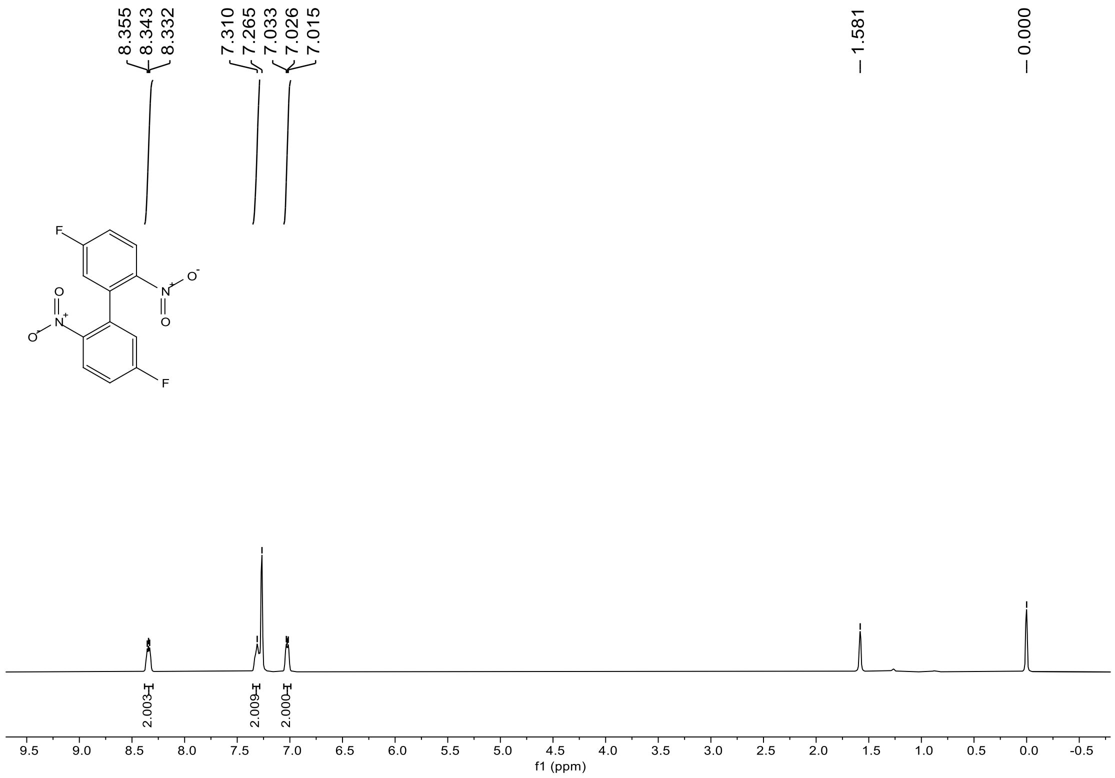

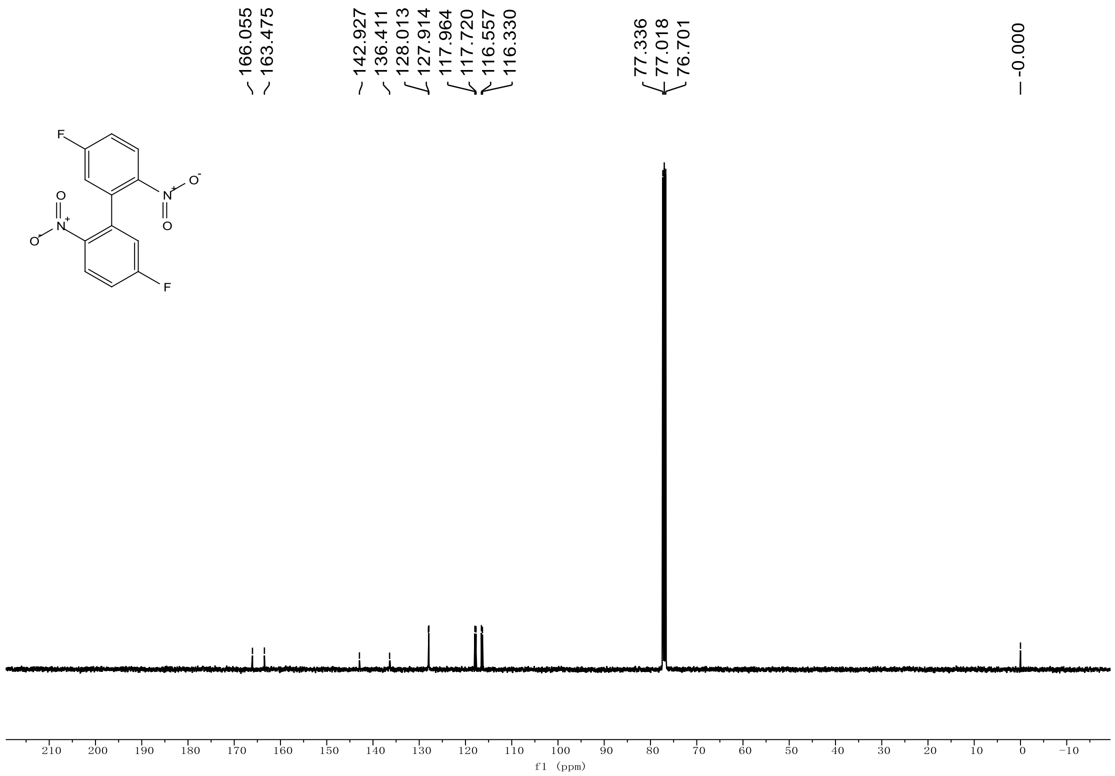
166.055
163.475
142.927
136.411
128.013
127.914
117.964
117.720
116.557
116.330
77.336
77.018
76.701
-0.000
F
O
N
f1 (ppm)

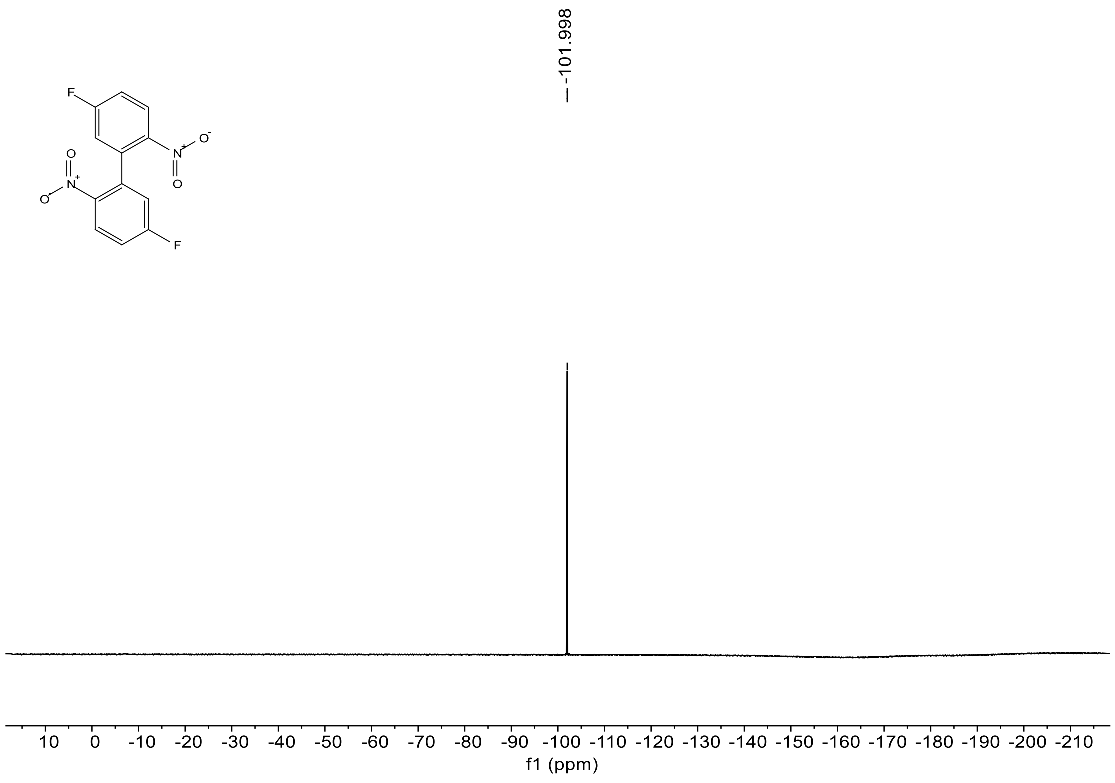
-101.998
F
O
N
f1 (ppm)

Figure S2 ^{1}H, ^{13}C and ^{19}F-NMR Compound 3

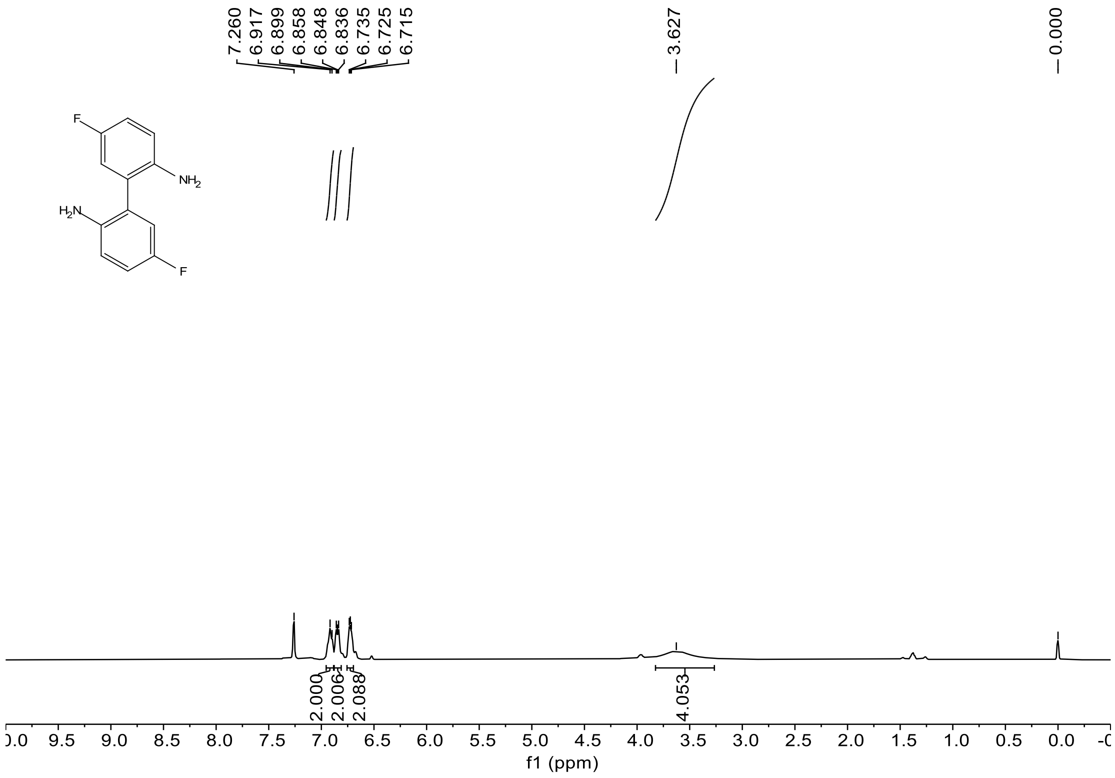

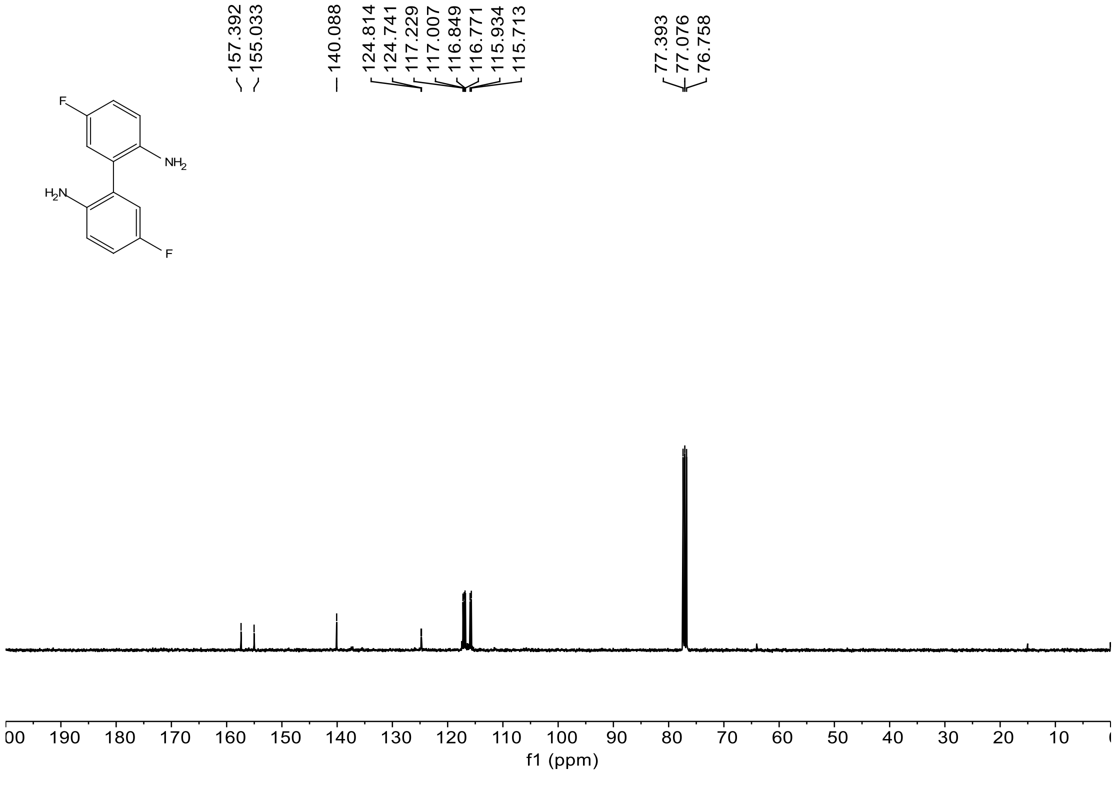

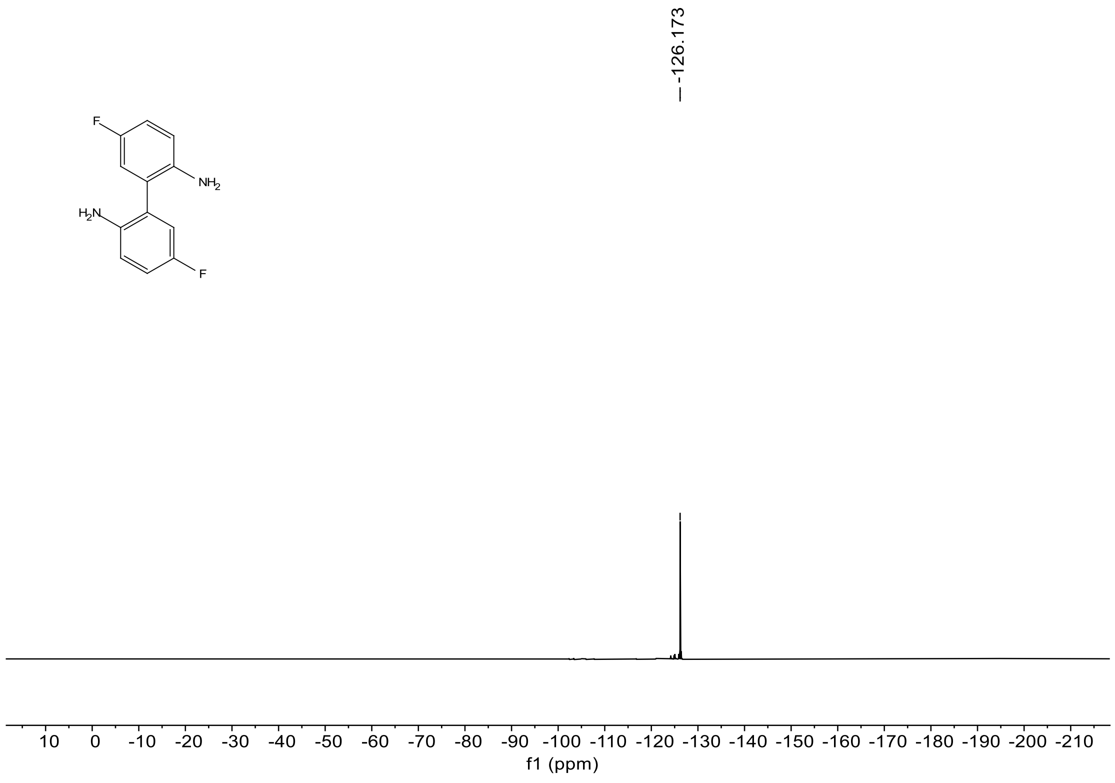

Figure S3 ^{1}H, ^{13}C and ^{19}F-NMR Compound 4

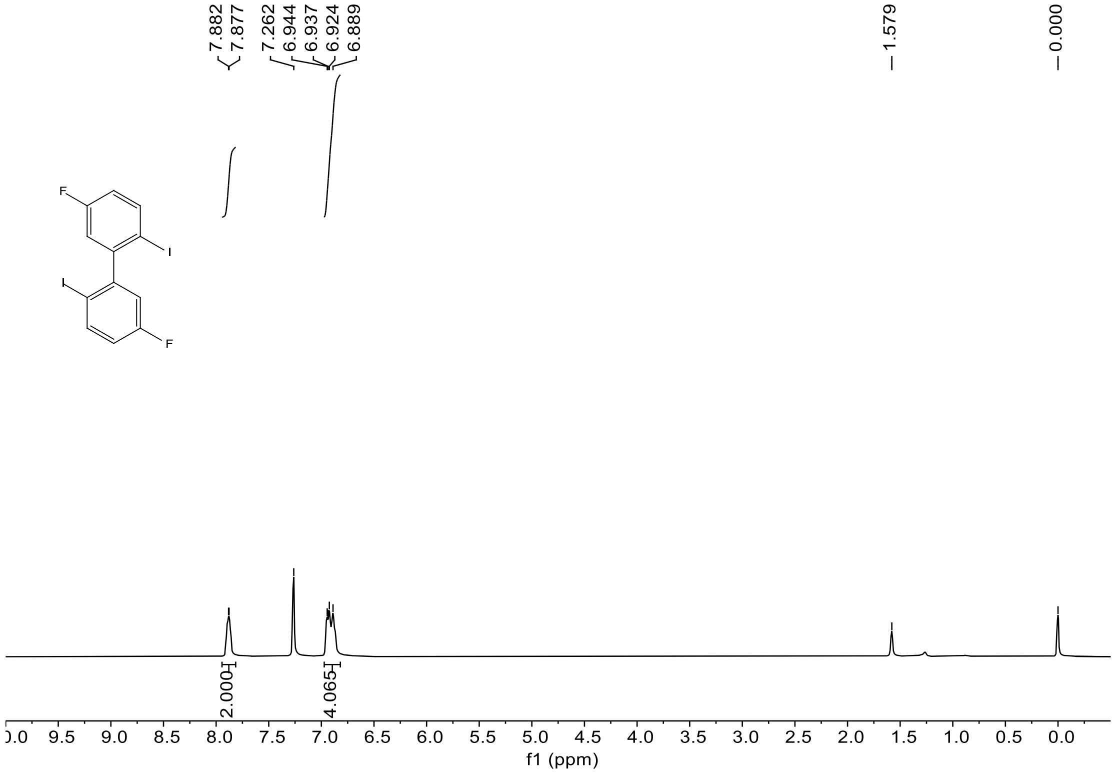

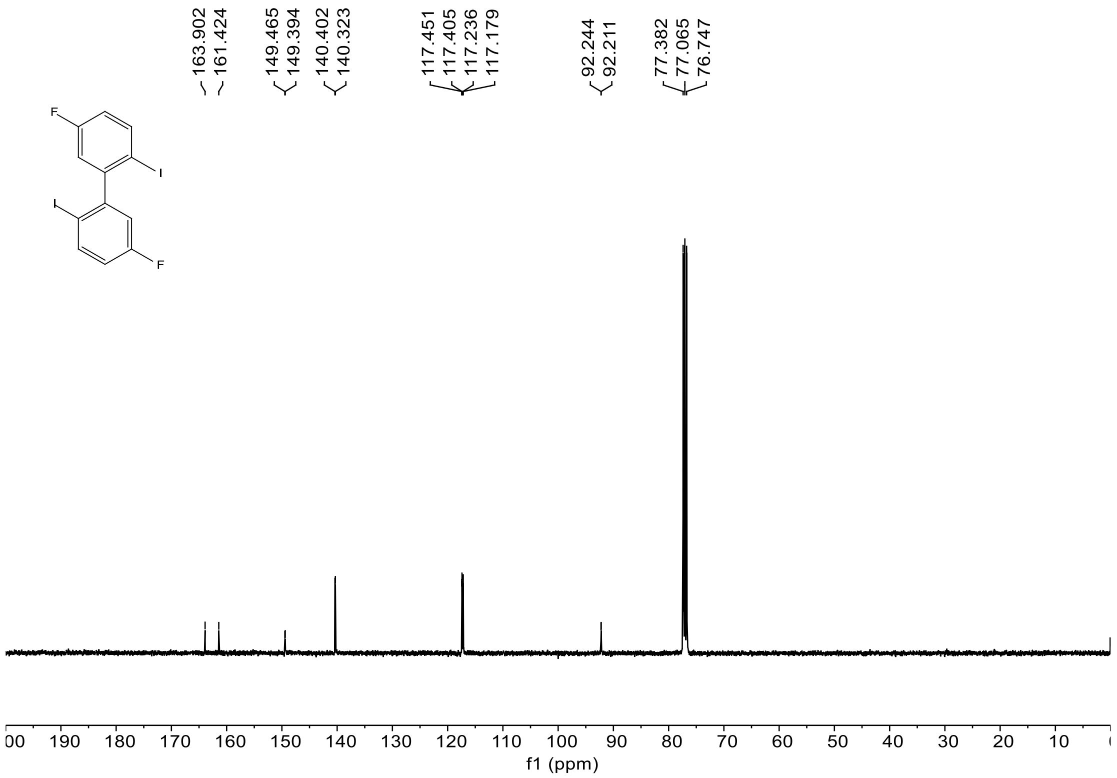
163.902
161.424
149.465
149.394
140.402
140.323
117.451
117.405
117.236
117.179
92.244
92.211
77.382
77.065
76.747
F
I
I
F
00 190 180 170 160 150 140 130 120 110 100 90 80 70 60 50 40 30 20 10
f1 (ppm)

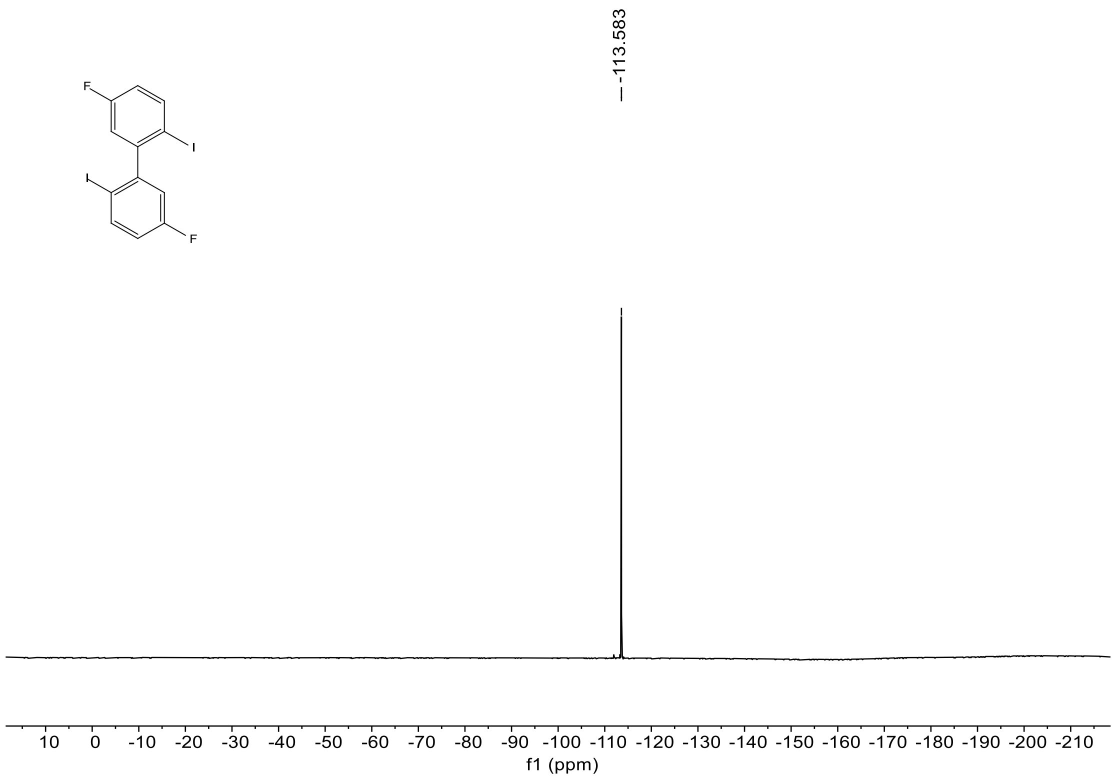
-113.583
F
I
I
F
10 0 -10 -20 -30 -40 -50 -60 -70 -80 -90 -100 -110 -120 -130 -140 -150 -160 -170 -180 -190 -200 -210
f1 (ppm)

Figure S4 ^{1}H, ^{13}C, ^{19}F and ^{31}P-NMR Compound 5

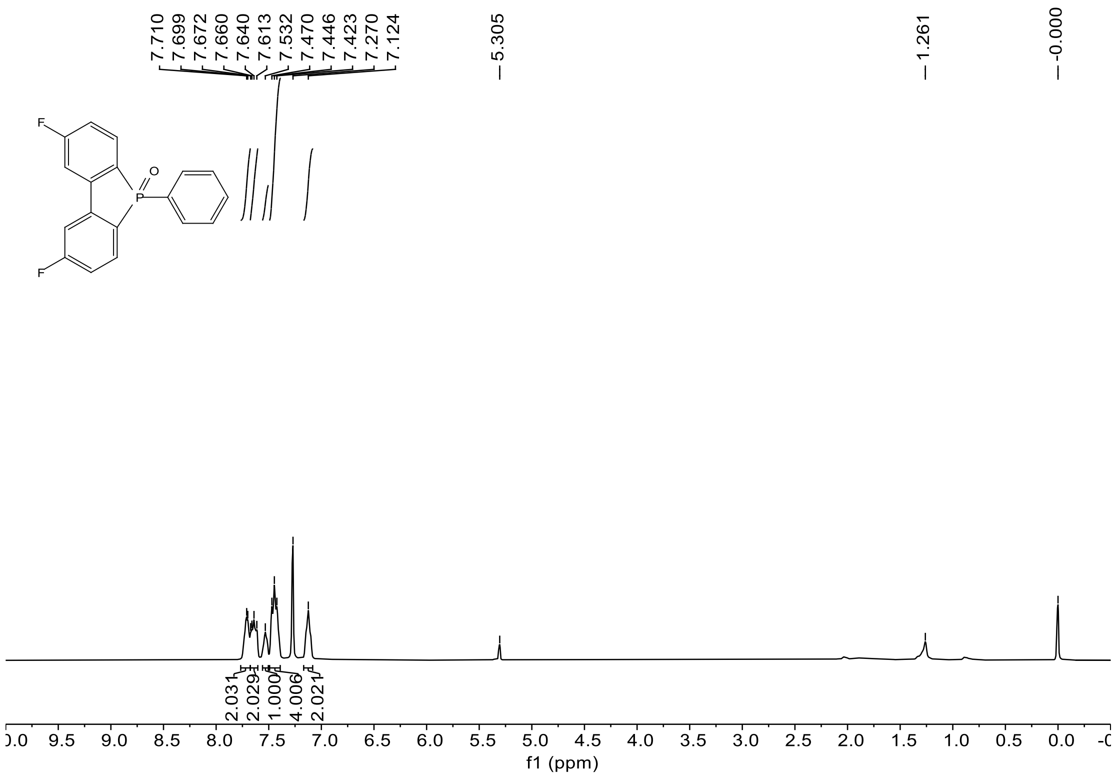

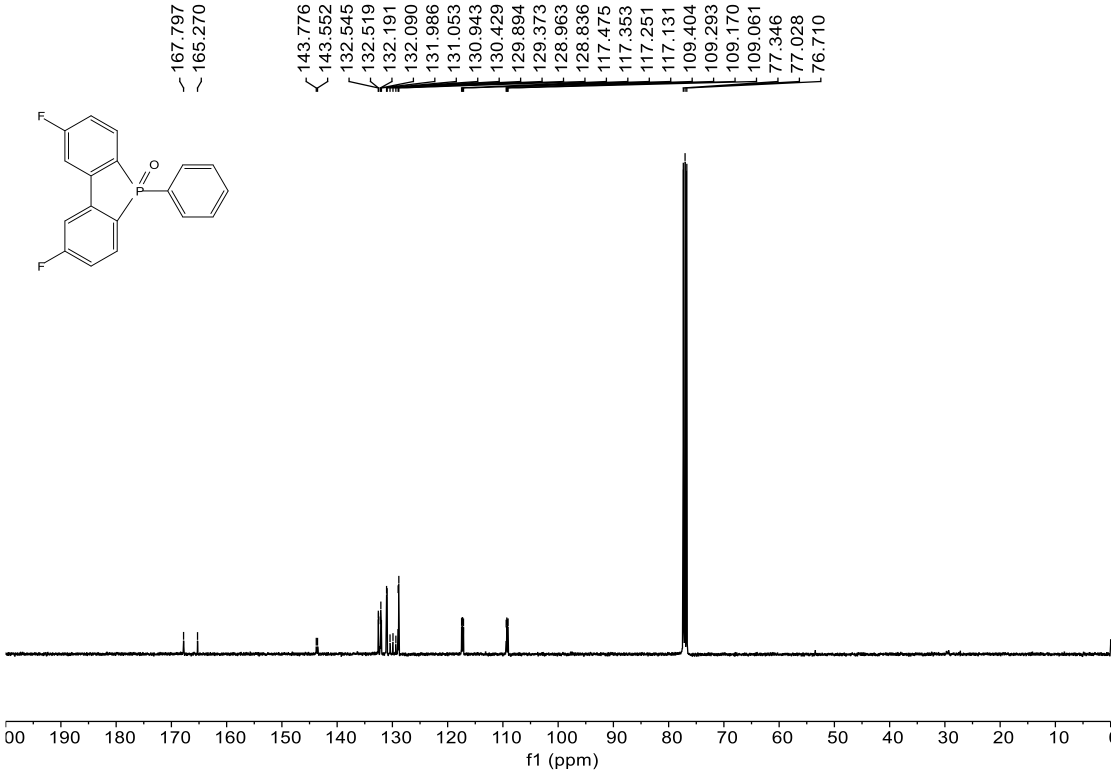

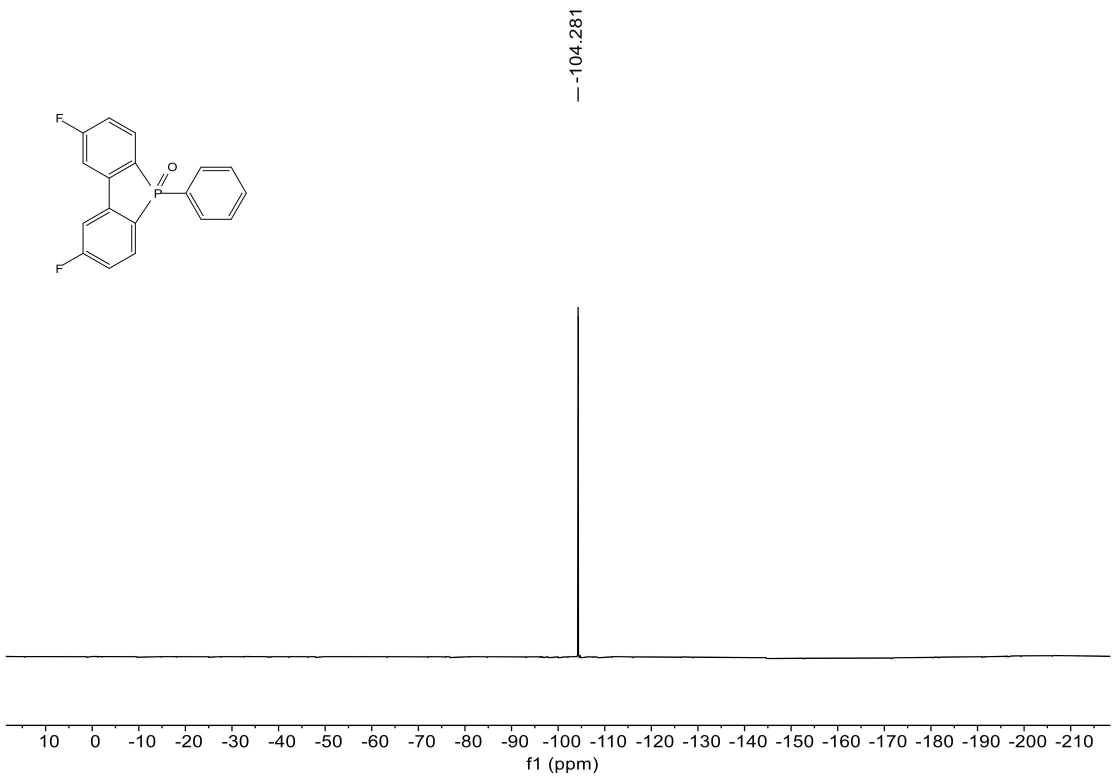
-104.281
F
F
O
P
10 0 -10 -20 -30 -40 -50 -60 -70 -80 -90 -100 -110 -120 -130 -140 -150 -160 -170 -180 -190 -200 -210
f1 (ppm)

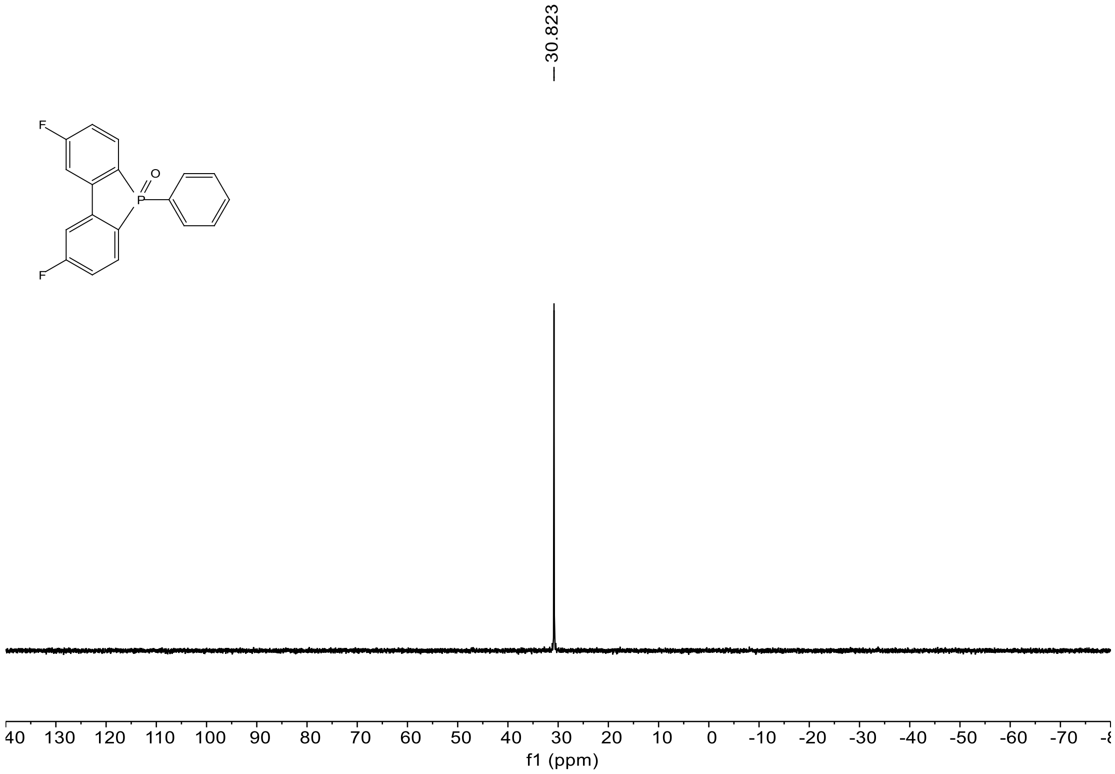
30.823
F
F
O
P
40 130 120 110 100 90 80 70 60 50 40 30 20 10 0 -10 -20 -30 -40 -50 -60 -70
f1 (ppm)

Figure S5 ^{1}H, ^{13}C and ^{31}P-NMR Compound 7-H

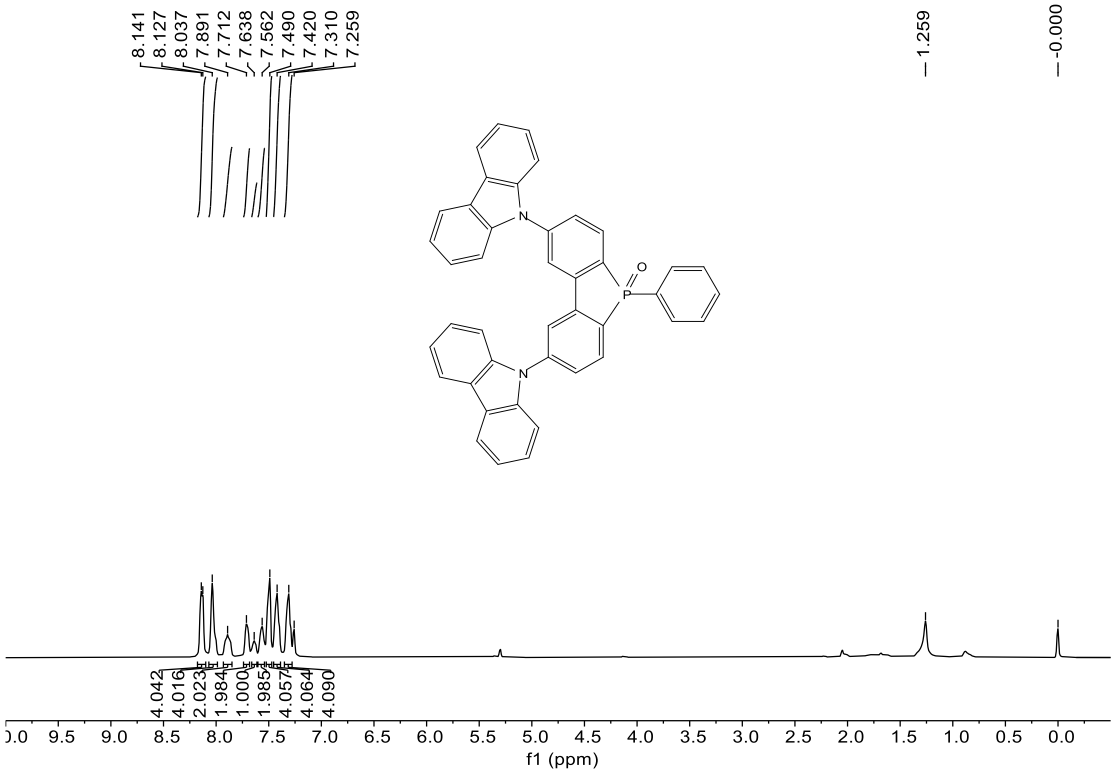

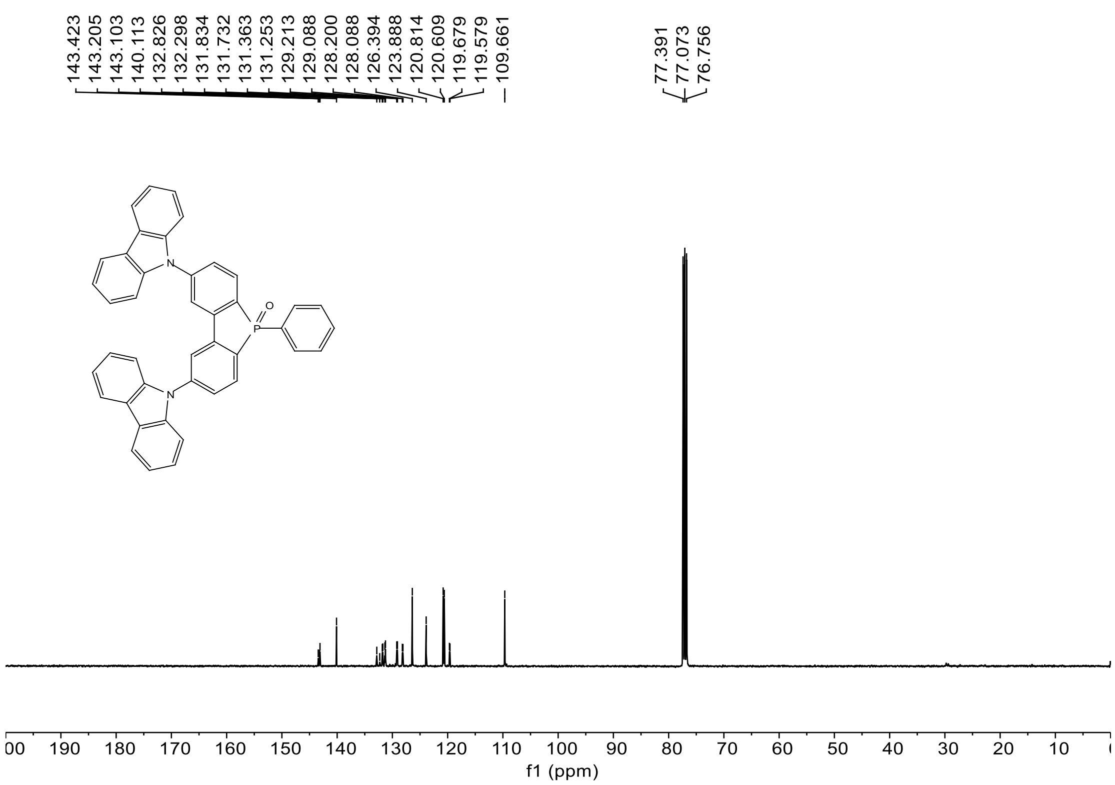

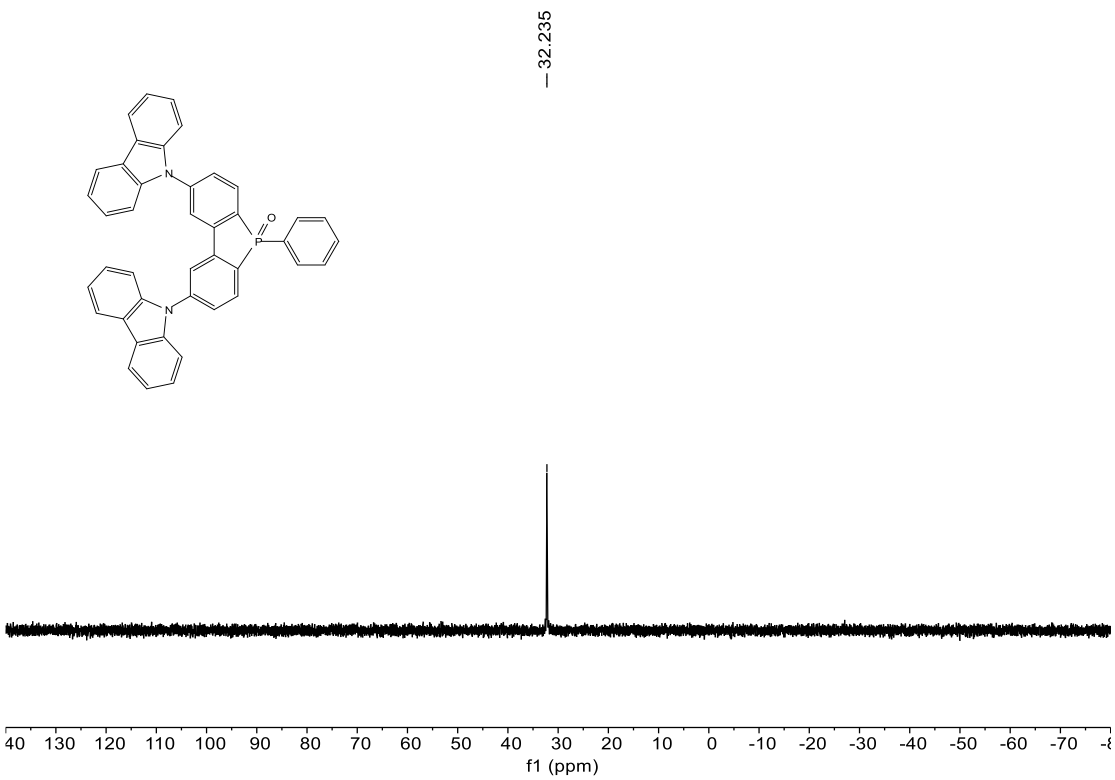

Figure S6 ^{1}H, ^{13}C and ^{31}P-NMR Compound 7-^{t}Bu

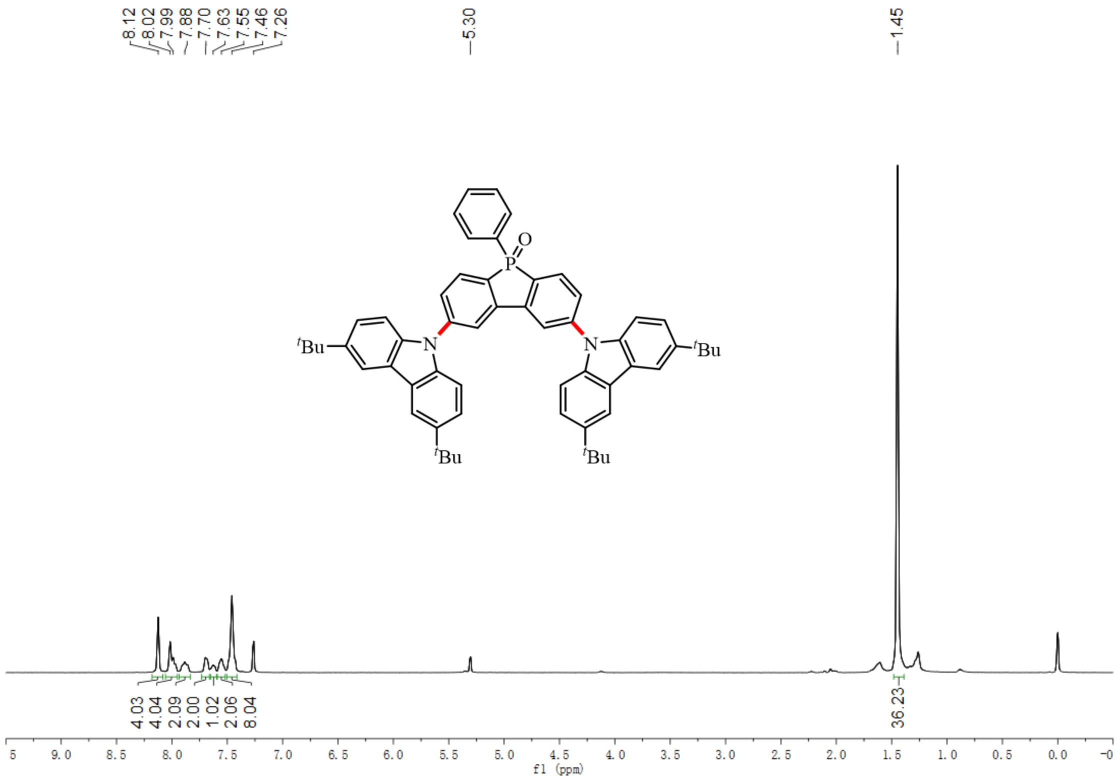

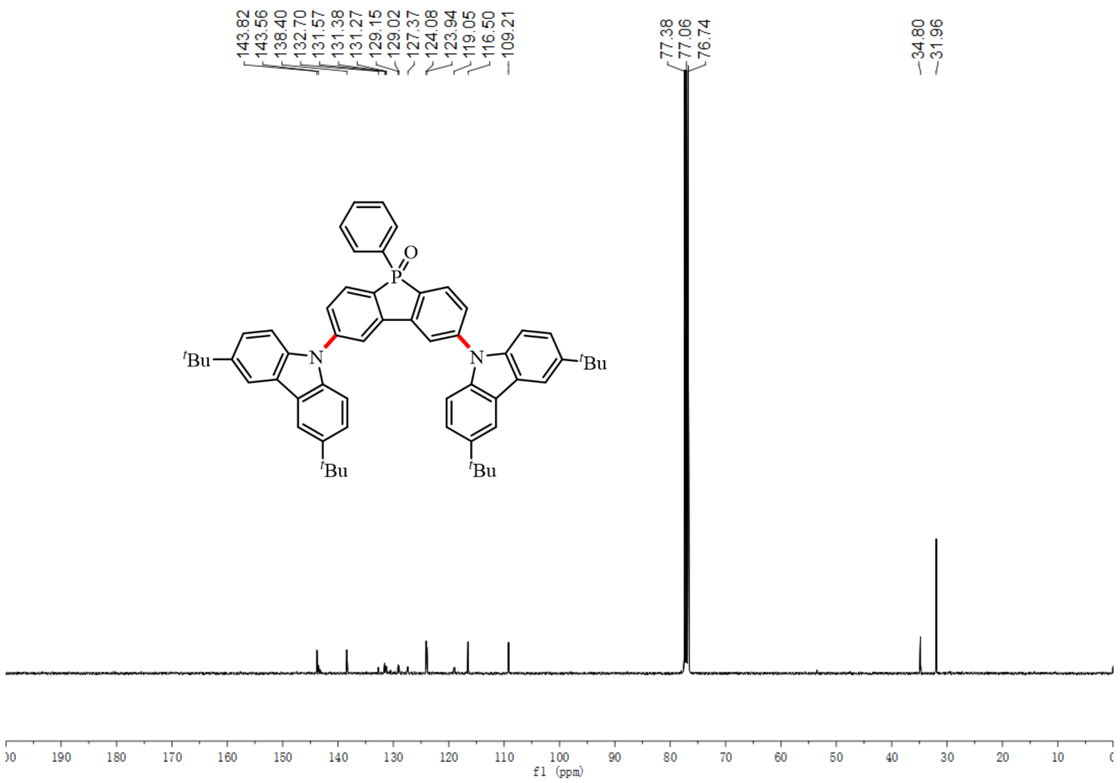
143.82
143.56
138.40
132.70
131.57
131.38
131.27
129.15
129.02
127.37
124.08
123.94
119.05
116.50
109.21
77.38
77.06
76.74
34.80
31.96
P
O
N
N
^{t}Bu
^{t}Bu
^{t}Bu
^{t}Bu
f1 (ppm)

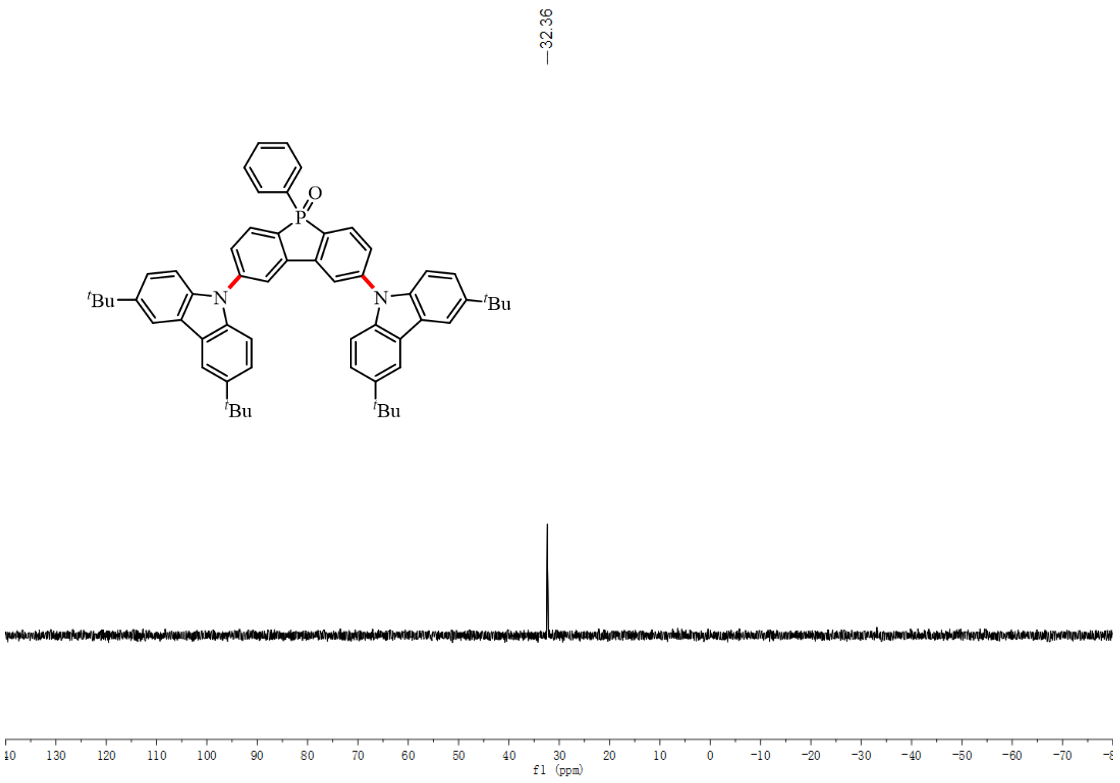
32.36
P
O
N
N
^{t}Bu
^{t}Bu
^{t}Bu
^{t}Bu
f1 (ppm)

Figure S7 ^{1}H, ^{13}C and ^{31}P-NMR Compound 7-Br

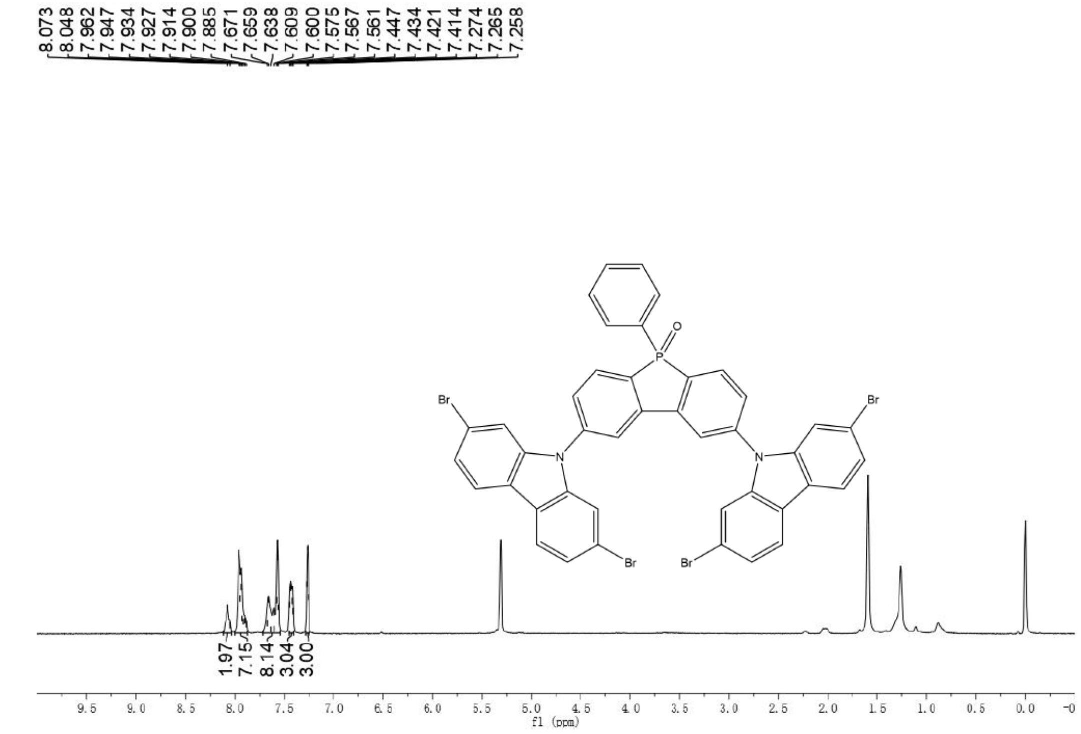

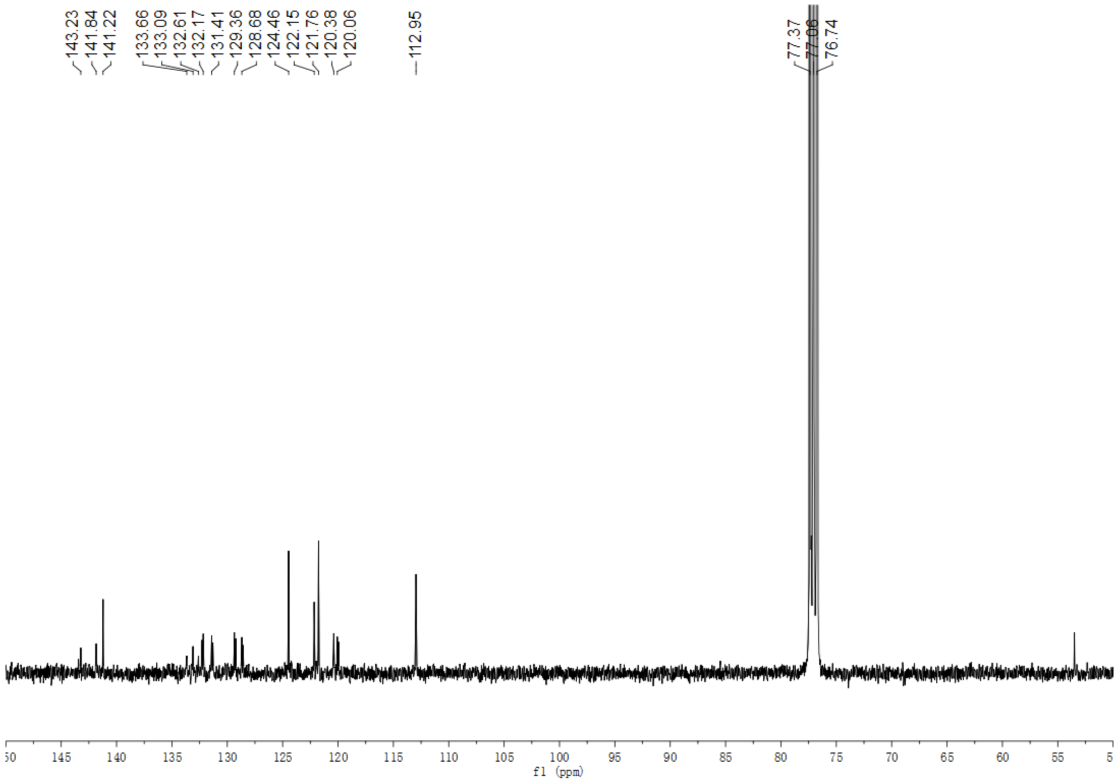

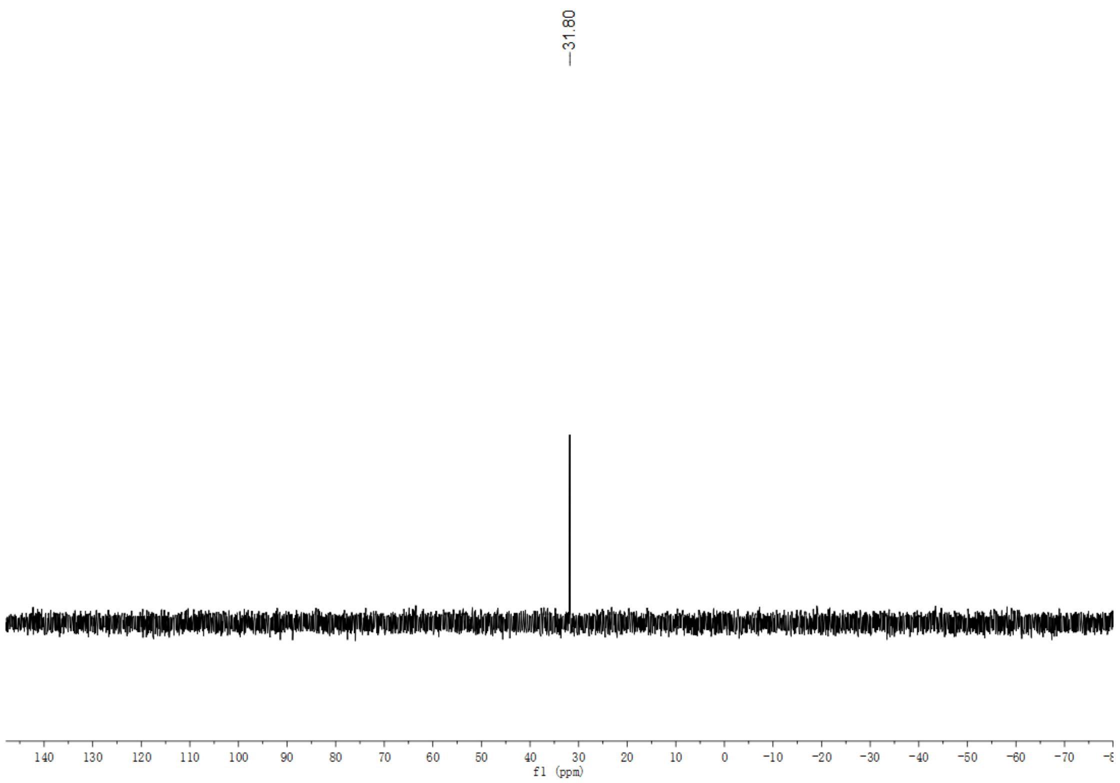

Figure S8 ¹H, ¹³C and ³¹P-NMR Compound 7-Cz-1

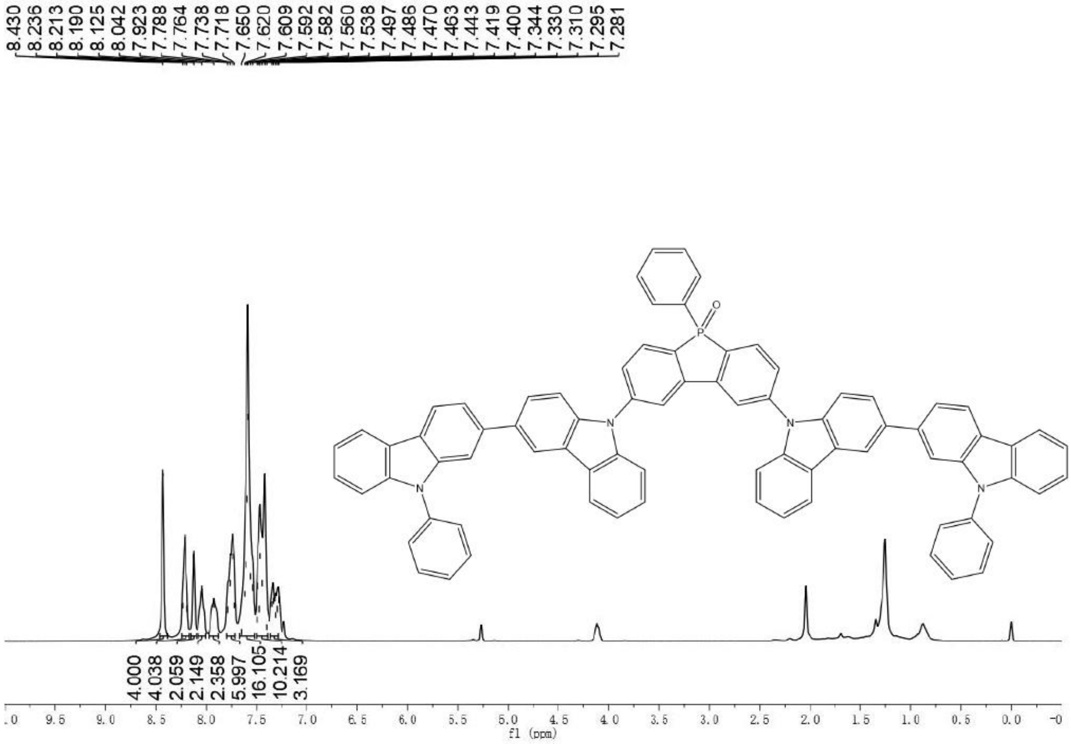

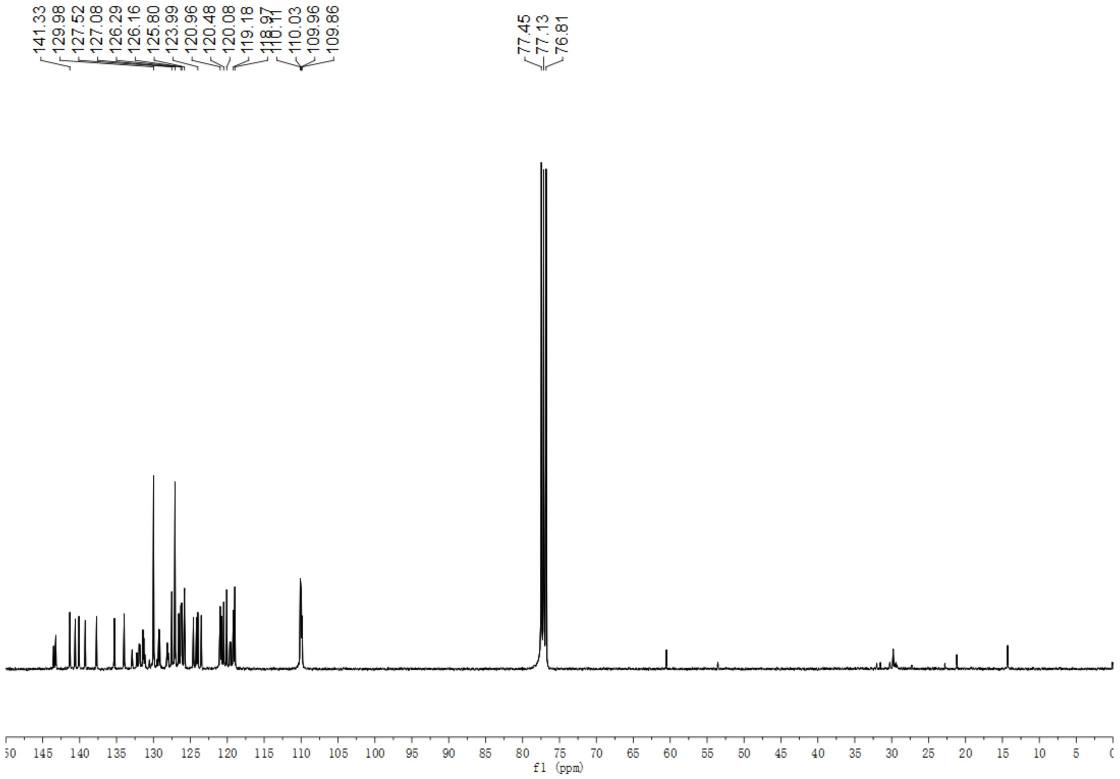
141.33
129.98
127.52
127.08
126.29
126.16
125.80
123.99
120.96
120.48
120.08
119.18
110.03
109.96
109.86
77.45
77.13
76.81
f1 (ppm)

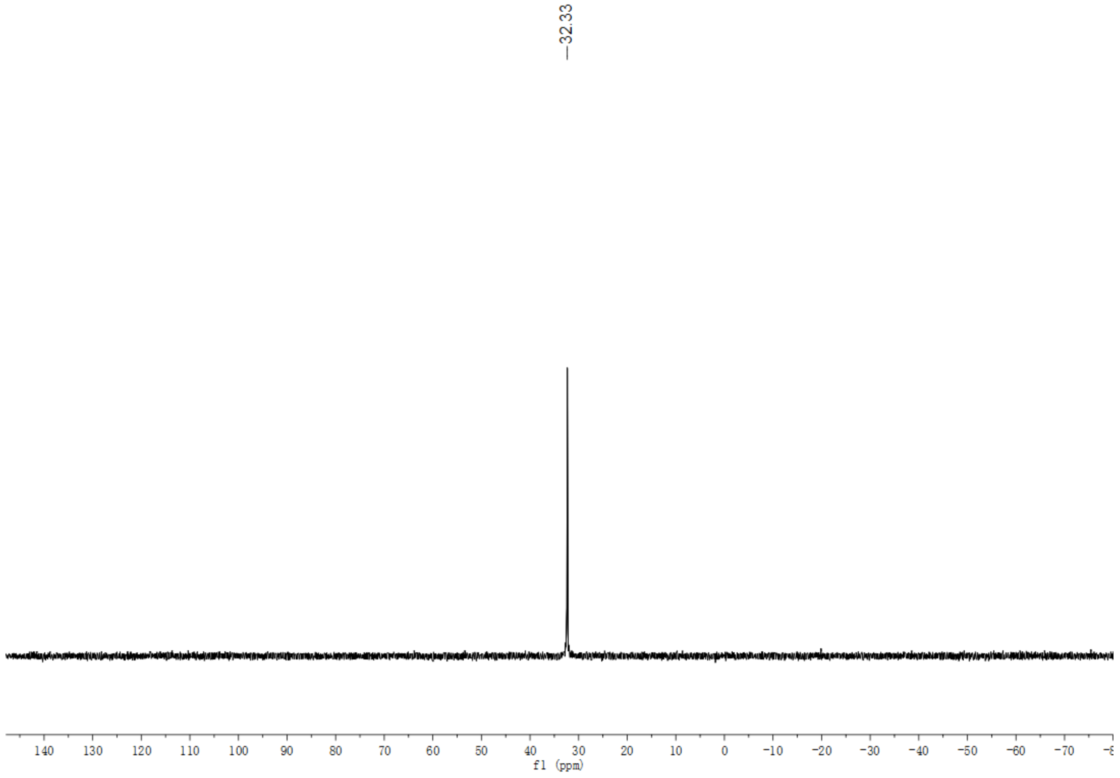
32.33
f1 (ppm)

Figure S9 ^{1}H, ^{13}C and ^{31}P-NMR Compound 7-Cz-2

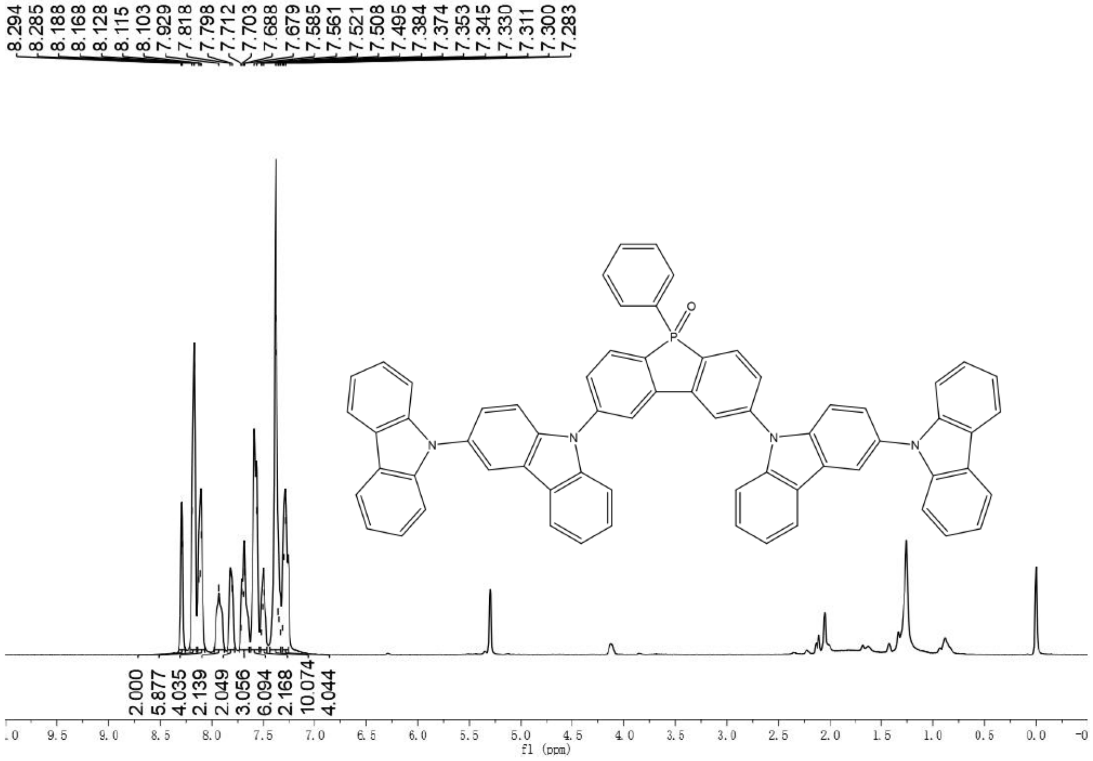

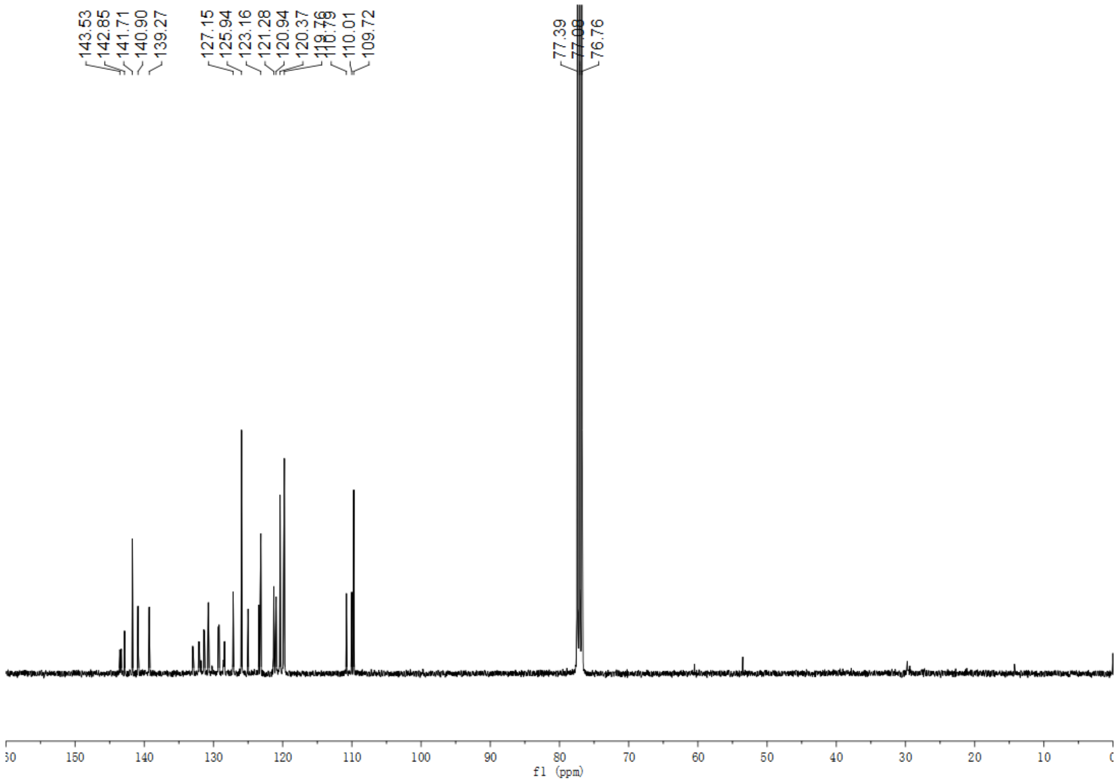

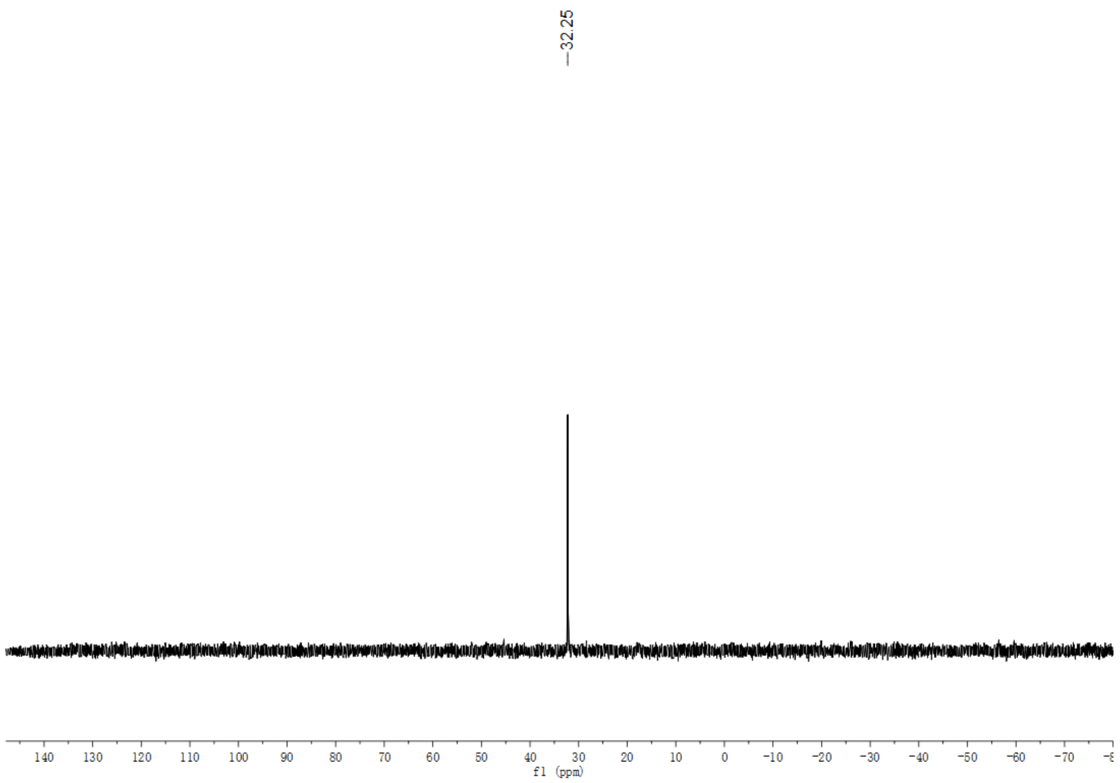

Figure S10 ^{1}H and ^{31}P-NMR Compound 7-Ph-1

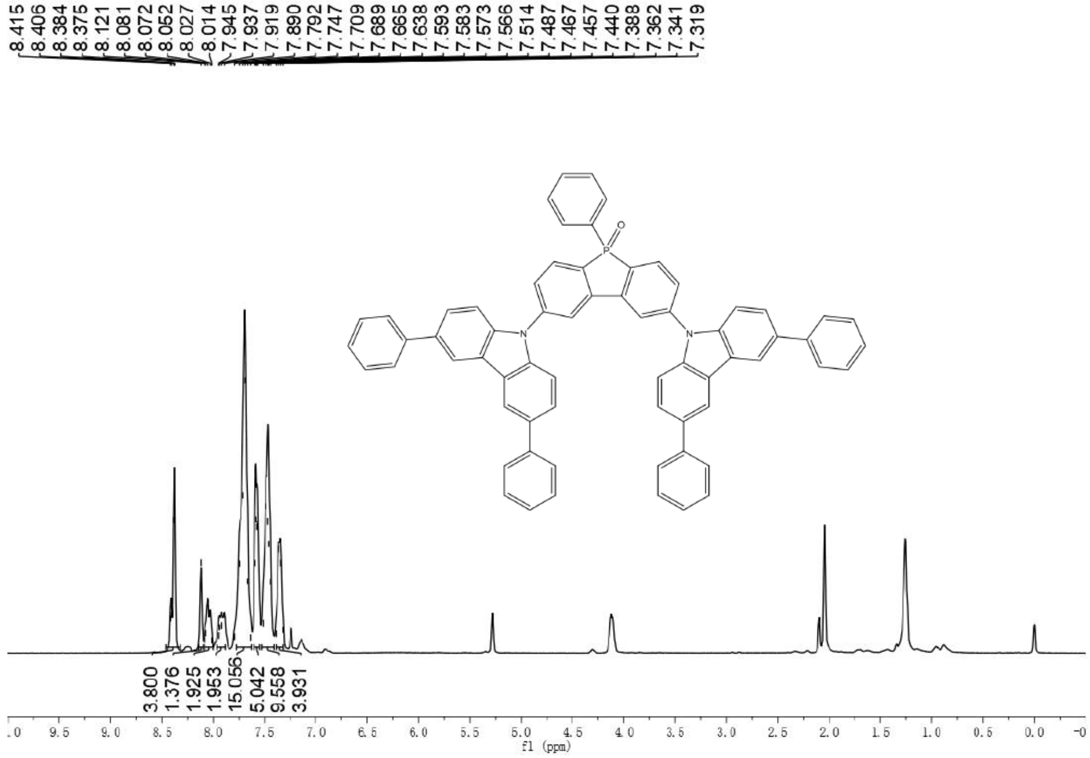

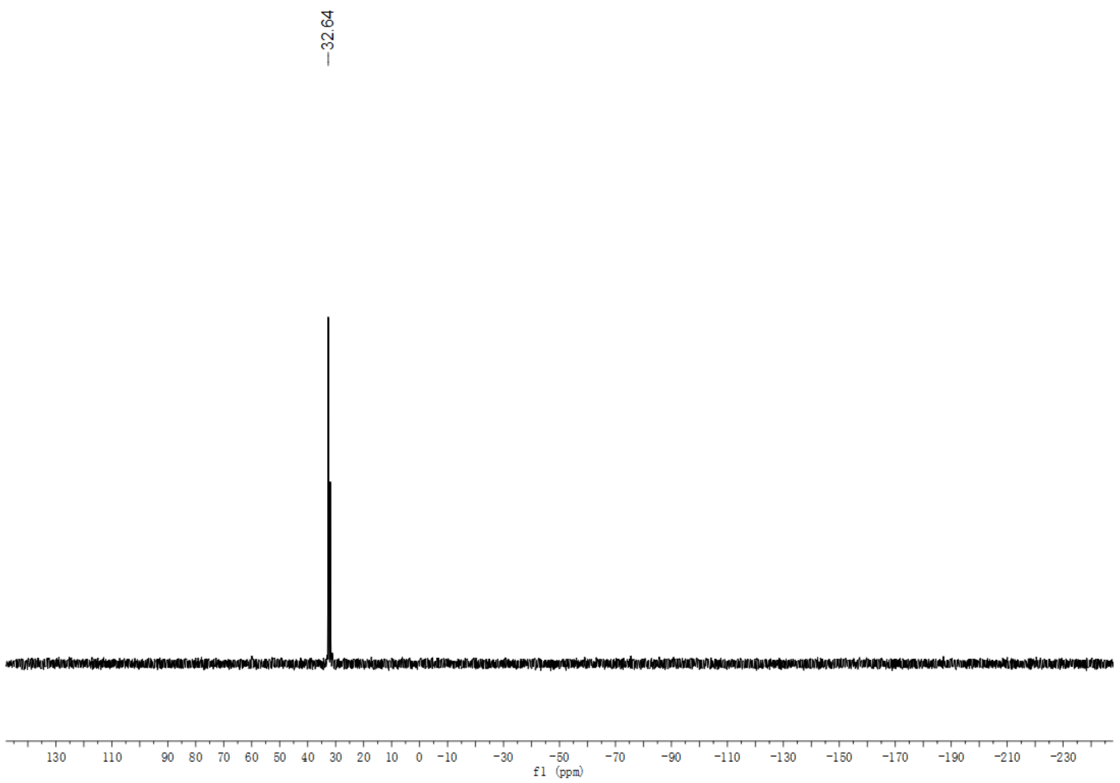

Figure S11 ^{1}H-NMR Compound 7-Ph-2

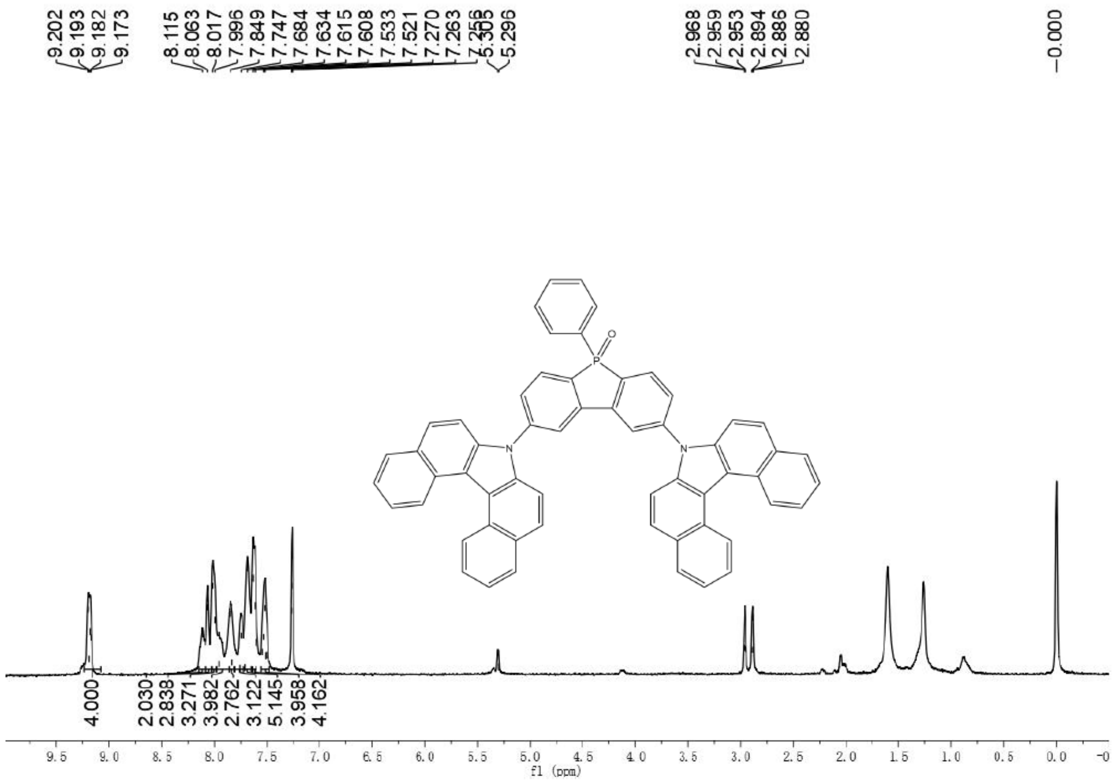

- **IR spectra**

Figure S12 7-H

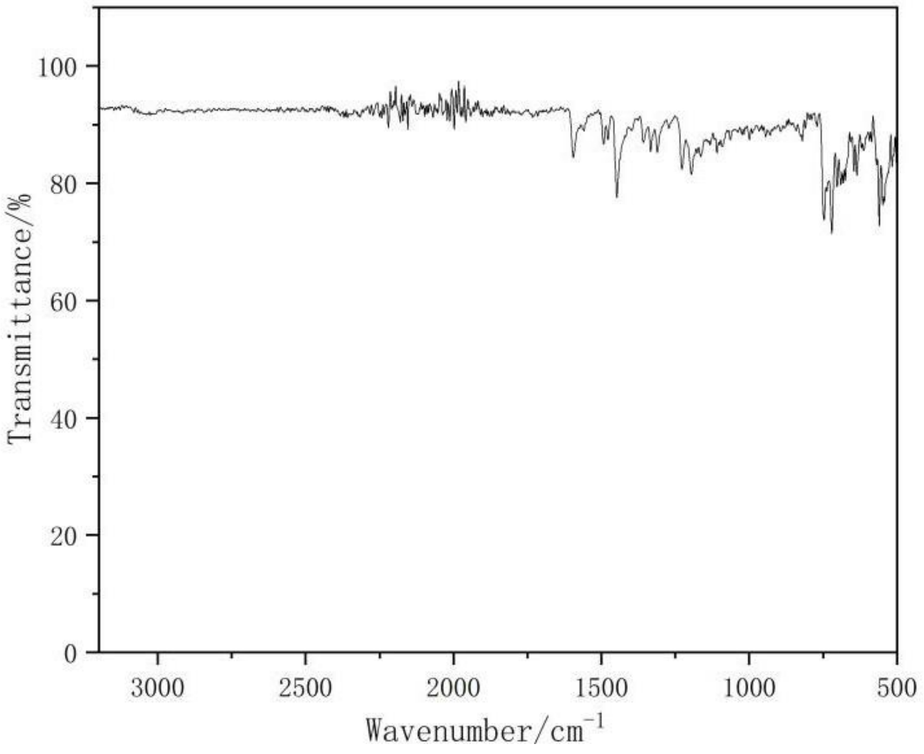

Figure S13 7-^{t}Bu

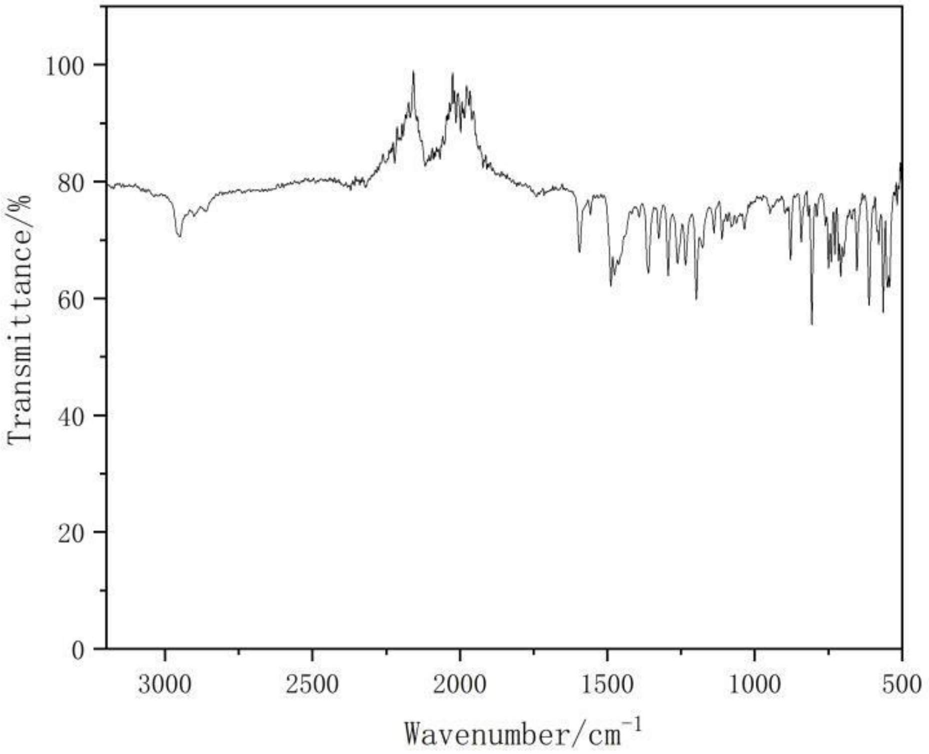

Figure S14 7-Br

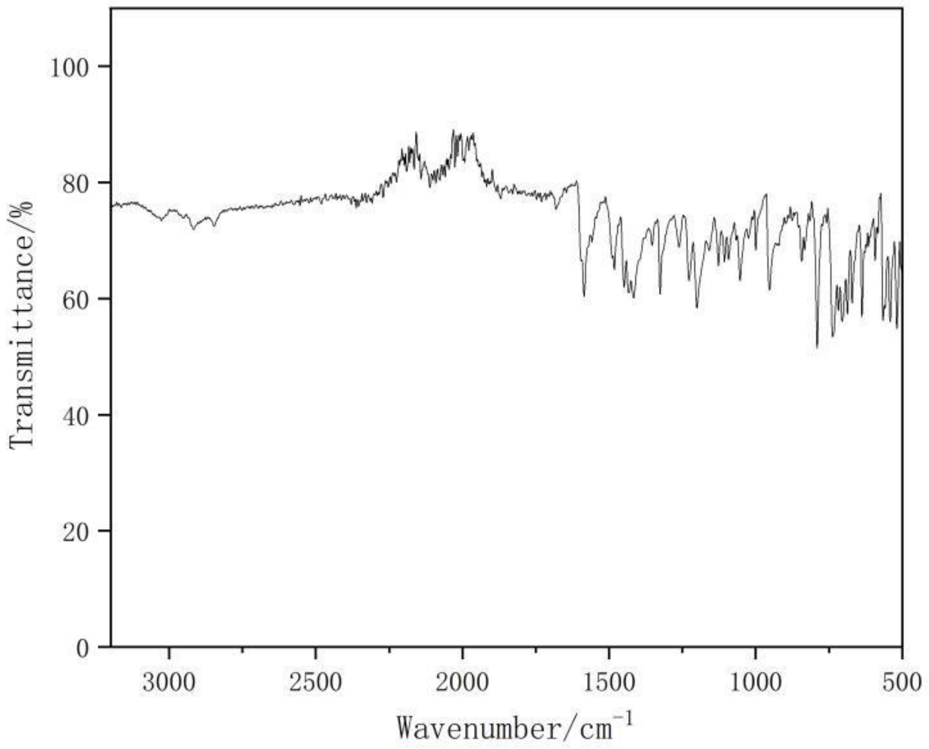

Figure S15 7-Cz-1

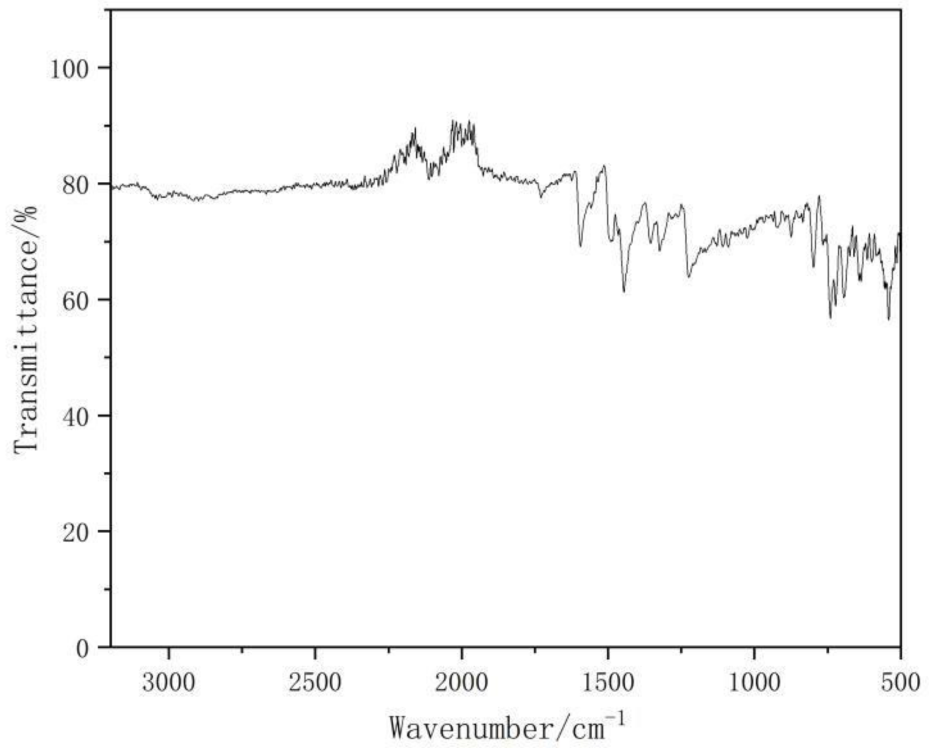

Figure S16 7-Cz-2

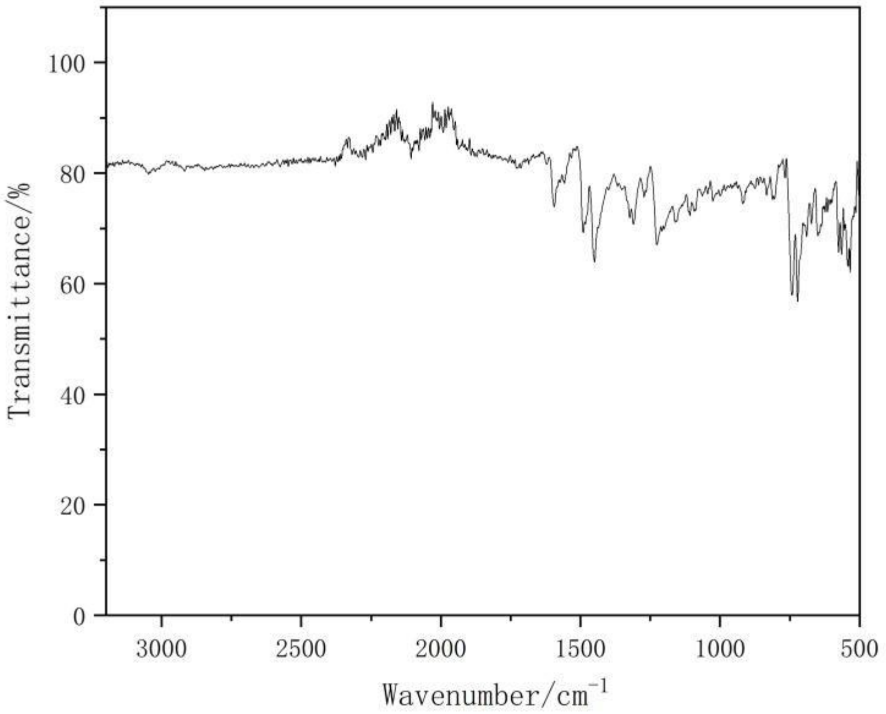

Figure S17 7-Ph-1

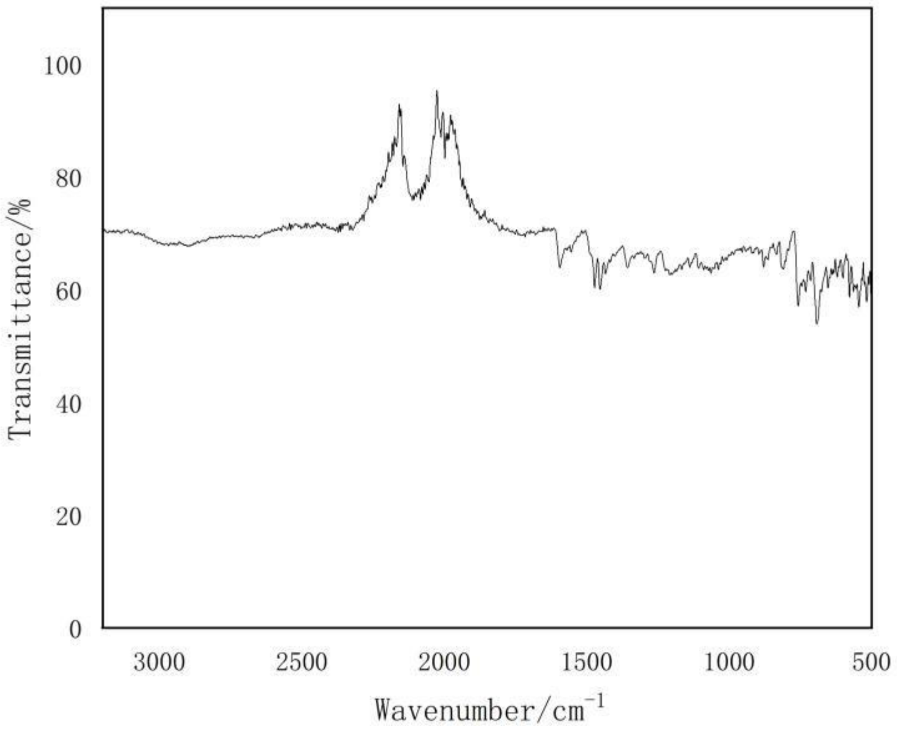

Figure S18 7-Ph-2

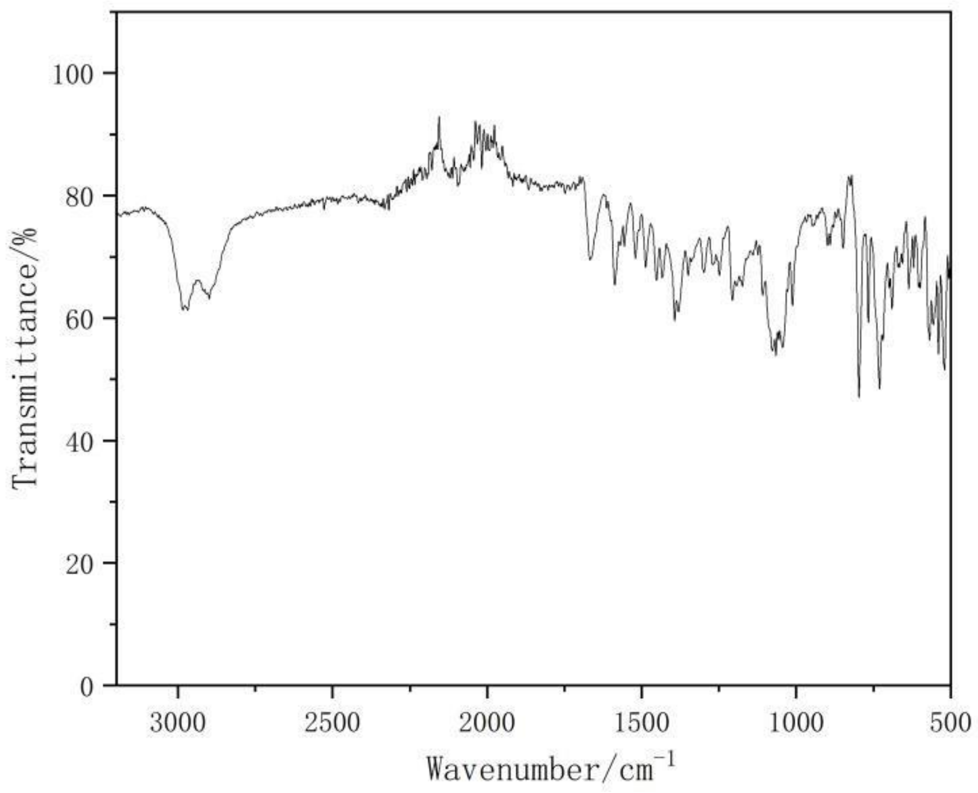

Supplement: File 1 — General information, experimental procedures, characterization data, and copies of spectra. [file Beilstein_J_Org_Chem-20-3299-s001.pdf]
